# Shared genome analyses of notable listeriosis outbreaks, highlighting the critical importance of epidemiological evidence, input datasets and interpretation criteria

**DOI:** 10.1099/mgen.0.000237

**Published:** 2019-01-16

**Authors:** Aleisha Reimer, Kelly Weedmark, Aaron Petkau, Christy-Lynn Peterson, Matthew Walker, Natalie Knox, Heather Kent, Philip Mabon, Chrystal Berry, Shaun Tyler, Lorelee Tschetter, Morganne Jerome, Vanessa Allen, Linda Hoang, Sadjia Bekal, Clifford Clark, Celine Nadon, Gary Van Domselaar, Franco Pagotto, Morag Graham, Jeff Farber, Matthew Gilmour

**Affiliations:** ^1^​ Public Health Agency of Canada, Winnipeg, MB, R3E 3R2, Canada; ^2^​ Health Canada, Bureau of Microbial Hazards, Ottawa, ON, K1A 0K9, Canada; ^3^​ Public Health Ontario, Toronto, ON, M5G 1M1, Canada; ^4^​ British Columbia Centre for Disease Control, Public Health Microbiology and Reference Laboratory, Vancouver, BC V5Z 4R4, Canada; ^5^​ Laboratoire de Santé Publique du Québec, Sainte-Anne-de-Bellevue, Québec, H9X 3R5, Canada; ^6^​ University of Guelph, Guelph, ON, N1G 2W, Canada

**Keywords:** clonal complex, genomic epidemiology, *Listeria monocytogenes*, listeriosis, outbreak, whole genome sequencing

## Abstract

The persuasiveness of genomic evidence has pressured scientific agencies to supplement or replace well-established methodologies to inform public health and food safety decision-making. This study of 52 epidemiologically defined *Listeria monocytogenes* isolates, collected between 1981 and 2011, including nine outbreaks, was undertaken (1) to characterize their phylogenetic relationship at finished genome-level resolution, (2) to elucidate the underlying genetic diversity within an endemic subtype, CC8, and (3) to re-evaluate the genetic relationship and epidemiology of a CC8-delimited outbreak in Canada in 2008. Genomes representing Canadian *Listeria* outbreaks between 1981 and 2010 were closed and manually annotated. Single nucleotide variants (SNVs) and horizontally acquired traits were used to generate phylogenomic models. Phylogenomic relationships were congruent with classical subtyping and epidemiology, except for CC8 outbreaks, wherein the distribution of SNV and prophages revealed multiple co-evolving lineages. Chronophyletic reconstruction of CC8 evolution indicates that prophage-related genetic changes among CC8 strains manifest as PFGE subtype reversions, obscuring the relationship between CC8 isolates, and complicating the public health interpretation of subtyping data, even at maximum genome resolution. The size of the shared genome interrogated did not change the genetic relationship measured between highly related isolates near the tips of the phylogenetic tree, illustrating the robustness of these approaches for routine public health applications where the focus is recent ancestry. The possibility exists for temporally and epidemiologically distinct events to appear related even at maximum genome resolution, highlighting the continued importance of epidemiological evidence.

## Data Summary

The data sets supporting the results of this article are included within the article and additional files. The draft and finished genomes are available at http://www.ncbi.nlm.nih.gov under BioProjects PRJNA167872–PRJNA167908, PRJNA215182–PRJNA215193, PRJNA36361 and PRJNA36363 (see Table S1, available in the online version of this article, for isolate-specific accession numbers).

**Impact Statement**

Implementation of whole-genome sequencing (WGS) technology for foodborne disease management is proceeding rapidly with continued development and refinement of interpretation guidelines, knowledge of limitations and development of informatics tools. Published WGS analyses of foodborne bacterial outbreaks to date are based on the analysis of raw WGS data. This study provides a thorough investigation of notable epidemiologically defined listeriosis outbreaks, based on manually annotated, closed and finished genomes, capturing all phylogenetic information through evolutionary modelling. This unique dataset, and companion epidemiological analyses, provides absolute inter- and intra-outbreak genetic distances and relationships to use as a calibration dataset for public health application, and as a benchmark dataset to test new bioinformatics tools. Our analysis of this dataset revealed three key observations: (1) our single nucleotide variant-based analytical approach is flexible and robust, yielding the same results despite variations in platform, read length, data quality, user and equipment – a necessary validation for decentralized surveillance systems such as those used for foodborne illness; (2) close genetic distances between epidemiologically related isolates are minimally affected by the size of the shared genome analysed; and (3) epidemiological support is paramount as WGS matches can be found between isolates known to have no epidemiological relationship.

## Introduction

Innovations in, and the cost-effectiveness of, DNA sequencing technologies have quickly tipped the scales to favour bacterial genome sequencing as the inevitable replacement of conventional subtyping for infectious disease surveillance and outbreak response. Public health laboratories now face the conceptual leap and technical challenges associated with the upheaval in well-established protocols, guidelines and expertise that have been the foundation of laboratory surveillance for decades [1, 2]. Due to the strength of existing listeriosis surveillance programmes and food regulatory activities associated with *Listeria* species, an international consortium of public health laboratories has chosen *Listeria monocytogenes* as a proof-of-concept pathogen for the application of whole-genome sequence (WGS) subtyping, bioinformatics analyses and inter-laboratory coordination of bacterial disease surveillance and response systems [3].

*L. monocytogenes* is the causative agent of listeriosis, a severe and often invasive illness comprising clinical manifestations of febrile gastroenteritis, bacteraemia and meningitis, with associated hospitalization and mortality rates of 60 % and 11–42 %, respectively [4, 5]. *Listeria* species are opportunistic pathogens ubiquitous in the natural environment, present in up to 5 % of healthy human carriers, and of particular concern to public health authorities and the food production industry as they are difficult to control using traditional food preservation means (salt and low temperatures). *Listeria*-contaminated food can result in large food product recalls, severe illness and high-profile fatal outbreak events [5–7].

The occurrence of outbreaks and sporadic listeriosis cases in Canada are relatively rare, with reported estimates of more than 200 domestically acquired cases per year in Canada (1600 annually in the United States) [8]. However, heightened surveillance and food safety control measures exist for *Listeria* species in both countries because of the high morbidity and mortality associated with listeriosis and because of the potential for significant impacts on the international trade of food commodities and overall health of food production economies. In other words, a small reduction in listeriosis cases saves lives and has a large impact on the food production industry and food safety regulatory activities [9].

Routine, real-time and integrated laboratory surveillance of listeriosis cases and *L. monocytogenes* is fundamental to public health and food safety systems at local to global scales. PulseNet Canada, a national network of regional public health and food safety laboratories and a founding member of PulseNet International, routinely subtype clinical, food and environmental isolates of foodborne bacterial pathogens using classical antigen serotyping and PFGE to detect subtype clusters/outbreaks and enable public health and food safety investigation and response activities. Other subtyping methods such as multilocus sequencing typing (MLST) and multiple-locus variable number tandem repeat analysis (MLVA) have also been applied as supplemental tools or to investigate the molecular epidemiology and evolution of bacterial pathogens including *L. monocytogenes* [10–12].

The vast majority of human listeriosis cases are caused by two of four defined evolutionary lineages of *L. monocytogenes* that correspond to traditional serotype groupings, with genetically distinct lineages I (serotypes 1/2b and 4b) and II (serotype 1/2a) largely differing in epidemiology and ecology [3]. *L. monocytogenes* population structure is further characterized into clonal complexes (CCs) using a globally recognized MLST scheme [10, 13]. Although the prevalence and epidemiology of disease-causing *L. monocytogenes* lineages and subclones often differs significantly by region, many outbreak-causing clones are globally widespread [14, 15]. Historically, lineage I strains have been responsible for the majority of severe disease and large outbreaks while lineage II strains have been observed mostly as sporadic clinical cases [3]. While this is still the case in some regions, many countries are observing a trend indicating the emergence of lineage II as a significant cause of clinical disease cases and outbreak events [6, 14, 16].

The world’s first listeriosis outbreak definitively linked to a food source occurred in eastern Canada in 1981 (sequenced in this study) [17]. Deaths of 17 pregnant women, fetuses and newborn babies were reported among a total of at least 41 confirmed outbreak cases exposed to coleslaw contaminated with a lineage I CC1 strain, and made from cabbage fertilized with diseased ovine faeces.

One of the largest listeriosis outbreaks on record occurred in Canada in 2008; nationally distributed ready-to-eat (RTE) delicatessen (deli) meats contaminated with a lineage II CC8 strain caused one of the deadliest listeriosis outbreaks, and initiated Canada’s largest outbreak and food safety investigations to date [5, 7]. Although the reported number of listeriosis cases had been on the rise since 2005, the magnitude of the 2008 event was unprecedented in Canada and resulted in at least 57 illnesses and a mortality rate of 42 % [2]. Notably, the subtype clone determined to cause the 2008 outbreak, CC8, in Canada is largely represented by common *L. monocytogenes* PulseNet Canada PFGE patterns LMACI.0001, LMACI.0040 and some highly similar variants [16]. Intriguingly, CC8 had commonly been recovered from food products and sporadic clinical cases, yet was rarely associated with outbreak events prior to 2008 [18] although lineage II, including CC8, was responsible for causing a significant proportion of sporadic human illnesses and smaller temporal case cluster events within Canada [18, 19].

Traditional subtyping methods have historically targeted genetic or phenotypic traits that evolve at a rate that balances the need for high discriminatory power while maintaining epidemiological concordance and have known resolution limitations in that they only capture a small proportion of the genetic information and are occasionally, and inherently, subject to subtype reversions [20]. Consequently, epidemiologically unrelated isolates are not always distinguished; this occasional low discriminatory power results in reporting of predominant, unresolved subclones within *L. monocytogenes* surveillance programs [14, 18, 19]. Subtype clones have a confounding effect on outbreak investigations, as contemporaneous isolates that have no true epidemiological or biological linkage may be indistinguishable if they have the same subtype designation [14, 21]. Highly prevalent subtype clones are also problematic for outbreak detection and defining outbreak limits as the endemicity of these clones limits the recognition and resolution of temporal subtype clusters as one cluster of disease cases drifts into the next, with no apparent lag between case clusters. In this capacity, traditional methods that achieve high discriminatory power such as PFGE and MLVA are subject to potential losses in epidemiological concordance when higher rates of evolution and/or horizontal gene transfer events obscure genetic relatedness between closely related strains involved in the same outbreak event.

WGS has already shown potential as a replacement subtyping tool for highly clonal organisms such as *Salmonella* Heidelberg where three outbreaks caused by indistinguishable and highly prevalent PFGE subtypes were easily distinguished using SNVPhyl [22, 23] as well as genetically diverse organisms such as *Escherichia coli* [24]. The latter study also indicates that WGS can provide faster resolution of subtype clusters than traditional methods such as MLVA – thus inclusion of WGS into case cluster definitions would improve efficiency by more accurately focusing investigative resources and uncomplicating food exposure analyses. We hypothesize that informative genomic diversity remains unsampled in conventional subtyping methods applied to subtype clones, and that this diversity could be uncovered with a comprehensive approach such as WGS to enhance strain discrimination for public health investigations. Furthermore, we hypothesize that the phylogenetic inferences based solely on vertically inherited markers can simplify the interpretation of outbreak events where convergent evolution via horizontal genetic transfer may be obscuring the genetic relatedness between isolates. Public health agencies around the globe have begun to implement genomic approaches to their routine listeriosis/*L. monocytogenes* surveillance and monitoring activities; it is now prudent and urgent to conduct comprehensive and validated surveys of the genomic diversity of both retrospective and contemporary foodborne disease isolates, including *L. monocytogenes*. These genomic epidemiological ‘calibrator studies’ using closed finished genome data and bioinformatics tools are the crucial first step to fully understand the power and limits of WGS and to inform data interpretation criteria and guidelines within the contexts of food safety and epidemiological investigations of infectious disease events [25].

## Methods

### Isolate selection and surveillance

*L. monocytogenes* isolates included in this study are listed in Table S1. Isolates from the Public Health Agency of Canada – National Microbiological Laboratory (PHAC-NML) culture collection representing Canadian historical listeria outbreaks occurring between 1981 and 2010 were selected for WGS. Specific emphasis was placed on selection of isolates from a nationwide 2008 RTE deli meat outbreak as this event represented the largest and most diverse listeriosis outbreak in Canada [5, 7]. Additional isolates were selected to provide a cross-section of lineage I and lineage II clinical, food and environmental isolates from provincial and multi-provincial outbreaks and smaller case clusters spanning over 30 years.

For isolate selection, representative isolates from human-derived *L. monocytogenes* received by PHAC-NML between 1998 and 2011 (*n*=1075) were included in the MLST clinical sample pool based on the source type/illness severity (declining order of preference: fetal–maternal > cerebrospinal fluid > blood > stool > other). In cases where two or more isolates were associated with mother and newborn/fetus, the latter isolate was selected. Isolates were identified as CC8 according to the following sequence type (ST) profiles: 8, 120, 232, 289, 292, 387, 551 (www.pasteur.fr/mlst; 2011-12-12). Isolates in this CC8 group (15.8 %, *n*=170) belonged to the following PFGE types: LMACI (0001, 0002, 0035, 0040, 0096, 0098, 0122, 0128, 0203, 0618); and LMAAI (0001, 0002, 0003, 0097, 0214, 0322, 0414).

### DNA extraction and culture conditions

Genomic DNA was extracted from cultures grown overnight in brain-heart infusion (BHI) medium at 37 °C with the Qiagen DNeasy Blood and Tissue Kit (Qiagen) or the Epicentre Metagenomic DNA Isolation Kit for Water (Epicentre Technologies) following the manufacturers' instructions.

### Serotyping and PFGE

Isolates included in this study were serotyped using antisera agglutination and multiplex PCR methods [10]. PFGE profiling was performed using restriction enzymes *Asc*I and *Apa*I according to PulseNet protocols and BioNumerics v6.5 [10].

### Multi-locus variable number tandem repeat analysis

Multiplex PCR of nine variable number tandem repeat (VNTR) regions was performed as described [11, 12] with modifications. Each 10 µl PCR consisted of 20 ng genomic DNA, 2.5 mM MgCl_2_ and 0.2 mM dNTPs (Roche), 1.5 U of Platinum Taq polymerase (Invitrogen) and HPLC purified primers (Biosearch Technologies) at the following concentrations: R1 reaction: LM2 (0.02 µM), LM8 (0.075 µM), LM10 (0.03 µM), LM11 (0.04 µM) and LMV09 (0.315 µM); R2 reaction: LM3 (0.0375 µM), LM15 (0.15 µM), LM23 (0.11 µM), LM32 (0.044 µM). Forward primers were labelled with FAM, HEX or CalR590. PCRs were run on an Applied Biosystems GeneAmp 9700 PCR system (Applied Biosystems) under the following conditions: 95 °C for 5 min; 35 cycles of 94 °C for 20 s, 50 °C for 20 s and 72 °C for 20 s; 72 °C for 5 min. Amplified PCR products were diluted 1 : 20 with sterile distilled H_2_O, and 1 µl of diluted product was mixed with 8 µl of Hi-Di Formamide (Applied Biosystems) and 1 µl of GeneFlo 625 ROX DNA size standard (Chimerx). Following denaturation (95 °C for 3 min), reactions were cooled to room temperature and analysed by capillary electrophoresis using an ABI 3130*xl* Genetic Analyzer (Applied Biosystems) and POP7 polymer (Applied Biosystems) at 60 °C for 30 min with an injection voltage of 1.6 kV and a run voltage of 15.0 kV. Peaks were manually analysed to determine the VNTR copy number within each amplified region according to PulseNet protocols (www.cdc.gov/pulsenet).

### Multiple locus sequence typing

MLST data were obtained through a combination of traditional PCR and *in silico* methods. For *in silico* MLST analysis, finished or draft *L. monocytogenes* genomes were used to query locus-specific blast+ databases populated with allelic sequences from the Institut Pasteur *L. monocytogenes* MLST database (www.pasteur.fr/mlst). Hits with 100 % identity and 0 % gaps were used to assign an allelic number to each locus, and novel alleles were confirmed by PCR. ST numbers were assigned according to established guidelines (www.pasteur.fr/mlst). Molecular MLST was performed as described [10] using modified primer sequences (Table S8) based on an alignment of 16 *L. monocytogenes* genomes (08–5578, 08–5923, 08–6997, 08–7669, 10–0933, 10–1046, 10–4754, 10–4758, 10–5026, 81–0861, 98–2035, 99–6370, Clip81459, EGD-e, F2365, HCC23). PCRs were performed at an annealing temperature of 50 °C; the amplicon was purified by ultrafiltration (Amicon Ultra-0.5 Centrifugal Filter Unit with Ultracel-30 membrane; Millipore) and Sanger-sequenced using Big Dye v.3.1 chemistry on an ABI3730XL sequencer with a 50 cm array (Applied Biosystems).

### Whole genome shotgun sequencing

WGS pyrosequencing (GS-FLX-Titanium) was performed according to the manufacturer protocols (Roche Diagnostics) yielding 8 kb paired-end reads. Illumina 2×100 paired-end WGS was performed on a GAIIx at Eurofins MWG Operon and/or locally using TruSeq DNA Sample Prep v2 and TruSeq SBS v5 kits (Illumina). These methods provided >99 % genome coverage at an average depth of >20× and >500×, respectively, for 52 genomes. In addition, paired-end Illumina data (2×250 cycles) were obtained using Nextera XT DNA Library Preparation and MiSeq Reagent V2 (500 cycles) kits on a MiSeq instrument according to the manufacturer's instructions (Illumina) yielding >100× coverage.

### Genome assembly

For 454-Illumina hybrid assemblies, pyrosequencing reads were assembled *de novo* (Newbler v2.5.3); Illumina-only assemblies were generated using GAIIx data and Edena v3.120626 [26]. Contigs were ordered using the Staden gap4 package [27] and ABACAS [28] using the closed genome most closely related to the sample as reference (08–5578 and 81–0861 for serotypes 1/2a and 4b, respectively). Genomes were closed using PCR and Sanger sequence-based bridging of contigs. For error correction, GAIIx reads were mapped using CLC Genomics Workbench v4.8. Single nucleotide variant (SNV) conflicts were identified with MUMmer v3 [29] and resolved manually and/or by PCR and Sanger sequencing in CLC Genomics Workbench.

### Genome annotation

Genomes were annotated using a custom version of GenDB as described [30]. Annotations from strain 81–0861 were manually curated and used to transitively annotate other genomes by blast n against all regions in each query genome (reciprocal best blast hits >80 % identity and >80 % high scoring segment pairlength). Following transitive annotation with 81–0861, a new representative genome (08–6997) was used to manually curate functional annotations not transitively annotated. This iterative process was carried out until all regions in all genomes were annotated in full.

### SNV calling and phylogeny analyses

The SNVPhyl pipeline [31] commit f132bf6 was used to call SNVs and provide a maximum-likelihood analysis. For this, Illumina data were mapped to a genomic reference using smalt v.0.7.0.1 (smalt_index: -k 13 –s 6; smalt_map: –f samsoft -r –1 -y 0.5) [32] and variants were called using FreeBayes (pvar 0; ploidy 1; min-mapping-quality 30; min-base-quality 30; min-alternate-fraction 0.75) [33] and SAMtools v0.1.18 (mpileup>=15) [34]. Variants identified using both methods were included for downstream analysis. Calls within potential problematic regions including dinucleotide variants, indels and repetitive regions, predicted phages and genomic islands were identified manually using MUMmer v3.23 [29] phast v6.7 [35] and IslandViewer v3 [36]. Shared SNVs (SNV loci present in all isolates of the population analysed) were merged into a single meta-alignment file for phylogenetic modelling using PhyML v.20120412 (GTR+G substitution model; best tree topology search; initial BioNJ tree) [37]. Branch support values were estimated using the approximate likelihood ratio test to generate maximum-likelihood phylogenies. For minimum spanning trees, all CC8 isolates were arbitrarily assigned to a pseudo-ST based on profiles identified using SNV results from the SNVPhyl pipeline. In total, 32 isolates were assigned to one of 27 pseudo-STs. Predicted relationships were visualized as minimum spanning trees using PHYLOViZ v3 [38].

## Results and discussion

In order to provide the foundation for the genomic epidemiology of *L. monocytogenes*, we surveyed and compared a broad selection of *L. monocytogenes* genomes relevant to public health in Canada and globally (Tables S1 and S7). Retrospective isolates were selected to cross-section the population structure implicated in epidemiologically well-characterized listeriosis outbreak events, as well as to conduct an unprecedentedly thorough genomic characterization of a predominant subtype clone in Canada, LMACI.0001, MLST ST 292 and related variants, referred to herein as CC8 (Table S1) [18, 19].

### Validation of hqSNV bioinformatics pipeline using different sequencing platforms and Sanger confirmation confirms robustness of analytical approach

Shared genome SNVs are frequently exploited genetic markers informatically harvested from WGS data to measure genetic distances and infer phylogenetic relationships [23, 24, 39, 40]; however, the robustness of variant calling approaches are rarely sufficiently validated with biological and informatic error rates are largely unknown [41]. Genetic relationships measured using our hqSNV bioinformatics pipeline were consistent between two redundant datasets from the same 39 isolates, acquired 4 years apart, utilizing different sequencing platforms, library preparation methods, operators and laboratories, and resulting varying read qualities (Tables S1, S2). Data set 1 was acquired in 2010 and for all 39 isolates included Roche 454 mate-pair and single end reads, and Illumina GAIIx 150 bp length paired and single end data acquired at a combination of NML and different contractor labs. All 39 isolates were resequenced at the NML in 2014 using the Illumina MiSeq platform (250 bp paired end) with minimum average coverage depth of 50×; this comprises data set 2. In 2010–2011, dataset 1 was used to close and finish all 39 isolates; this closed finished dataset is referred to as dataset 3. Phylogenetic tree topology and genetic distances between outbreak-related isolates were consistent between datasets 1 and 2 (Fig. S1). Genetic distance values between highly related isolates were additionally supported by Sanger-confirmed SNVs in the closed finished genome data (data set 3), further validating the robustness of our hqSNV bioinformatics approach. Our finding supports smaller previous studies concluding that sequencing technologies and read quality are of little consequence in the application of WGS for distinguishing disease outbreaks with moderate genetic similarity [42], although such technical robustness has not been previously validated for highly clonal groups as is reported in this study.

Overall, hqSNV results indicate that robust informatics can moderate the effect of non-standardized input datasets of varying quality, platform origin and file format inputs for both diverse and highly clonal datasets. Despite these promising results, larger scale testing of these hypotheses and validation of our bioinformatics approach for other bacterial pathogens, development of data quality and informatics standards, and inter-laboratory validations are required before next-generation sequencing (NGS) can fully replace conventional subtyping methods in public health and food safety laboratories.

### Effect of genomic diversity on shared genome size and phylogenomic discriminatory power

The scope of diversity of the input strains set did not significantly alter the genetic distances measured between related isolates (as would be expected by a shared genome region defined by the input dataset) (Table 1), nor the topology of the phylogenetic trees. This observation was relevant to both highly divergent outbreak strains (in the context of the overall disease-causing *L. monocytogenes* population structure) as well as outbreak strains belonging to highly clonal groups of *L. monocytogenes*, an observation that further supports the suitability of our SNVPhyl bioinformatics pipeline and approach for the investigation of listeriosis outbreaks and shows promise for other bacterial pathogens of diverse or clonal population structure.

**Table 1.** Intra-outbreak typing comparison of *Listeria monocytogenes* Comparison of conventional subtyping results (serotyping, PFGE, MLST, MLVA) and genome analyses (whole genome comparison and shared hqSNV analyses based on species, lineage and CC shared genomes). These methods were applied to retrospective listeriosis outbreaks (*n*=9) and assessed for their ability to discriminate isolates with no known epidemiological relationship.

**Outbreak**	Serotype	LMACI PFGE	CC/MLST	MLVA	Whole genome analysis*	Species-specific hqSNVs	Lineage-specific hqSNVs	CC8-specific hqSNVs	Epidemiological link
1981 Coleslaw (*n*=4)									
	4b	3+ variants	CC1: ST1	15_4_5_5_4_1_3_16_18	~50–100 SNVs	12–18	11–17		
2002 Cheese A (*n*=3)									
	4b	0023	CC1: ST1	15_4_5_5_4_1_3_16_18	3 SNVs (in one internalin gene)	0	0		
2002 Cheese B (*n*=2)									
	4b	0082	ST388	15_3_5_5_2_1_3_7_21	0 SNVs	0	0		
1996 Imitation crab (*n*=2)									
	1/2b	0046	CC5: ST5	16_3_3_5_4_3_4_16_15	1 SNV	1	1		
2000 Whipping cream (*n*=2)									
	1/2a	0118	CC7: ST7	15_3_5_N_1_1_2_10_13	1 SNV	0	1		
2008 Cheese D (*n*=2)									
	1/2a	0149	CC415: ST394	17_3_5_3.5_1_1_2_19.5_13	0 SNVs	0	0		
2002 Cheese C (*n*=2)									
	1/2a	0616	CC37: ST37	18_3_5_N_1_4_2_12_13	1 SNV	1	1		
2008 Deli meat (*n*=10)									
	1/2a	0040	CC8: ST292	17_3_5_N_1_6_2_20_13	0–2 SNVs: Strain 1 (main outbreak strain) Clinical cases (*n*=2), FPE/food (*n*=3)	0–2	0–2	0–2	Deli meat exposure
	1/2a	0001	CC8: ST292	17_3_5_N_1_6_2_20_13	Strain 2 : 10–0814 (deli meat) (10 SNVs, ∆LMC1)	n/a (1–2)	n/a (1–2)	n/a (1–2)	
	1/2a	0001	CC8: ST292	17_3_5_N_1_6_2_20_13	Strain 3 : 08–7374 (deli meat) (25 SNVs, *buk*fs, ∆*gltX*, LMC1HGT)	n/a (3–4)	n/a (3–4)	n/a (3–4)	
	1/2a	0001	CC8: ST120	17_3_5_N_1_6_2_20_13	0 SNVs: Strain 4 (FPE isolates, *n*=3) (46 SNVs, *buk*fs, *gltX*, ∆LMC1)	0 (14–15)	0 (18–19)	0 (20–21)	
	1/2a	0001	CC8: ST120	17_3_5_N_1_6_2_20_13	Strain 5 : 08–7669 (clinical) (53 SNVs, *buk*fs, ∆*gltX*, ∆LMC1, ∆LMC2)	n/a (21–22)	n/a (26–27)	n/a (27–28)	No deli meat exposure
	1/2a	0001	CC8: ST120	17_3_5_N_1_6_2_20_13	Strain 6 : 08–5923 (clinical) (64 SNVs, *buk*fs, ∆*gltX*, ∆LMC1, LMC1HGT)	n/a (19–20)	n/a (22–23)	n/a (27–28)	
2010 Prosciutto ham (*n*=4)									
	1/2a	0001	CC8: ST120	17_3_5_N_1_6_2_20_13	0–5 SNVs (70 SNVs, *buk*fs, ∆*gltX*, ∆LMC1, LGI1HGT)	0–3 (16-18)	0–5 (20-22)	0–5 (22-24)	

*Mauve/Staden comparison of complete (closed and finished) genomes (exception: strain 4 FPE draft genomes); black font, intra-outbreak/intra-strain comparison; blue font, inter-strain comparison (to strain 1); HGT, horizontal transfer event (recombination/replacement); fs, frame shift; *∆*, deletion; LMC, putative bacteriophage-related coding sequences; FPE, food processing environment.

**Fig. 1.**
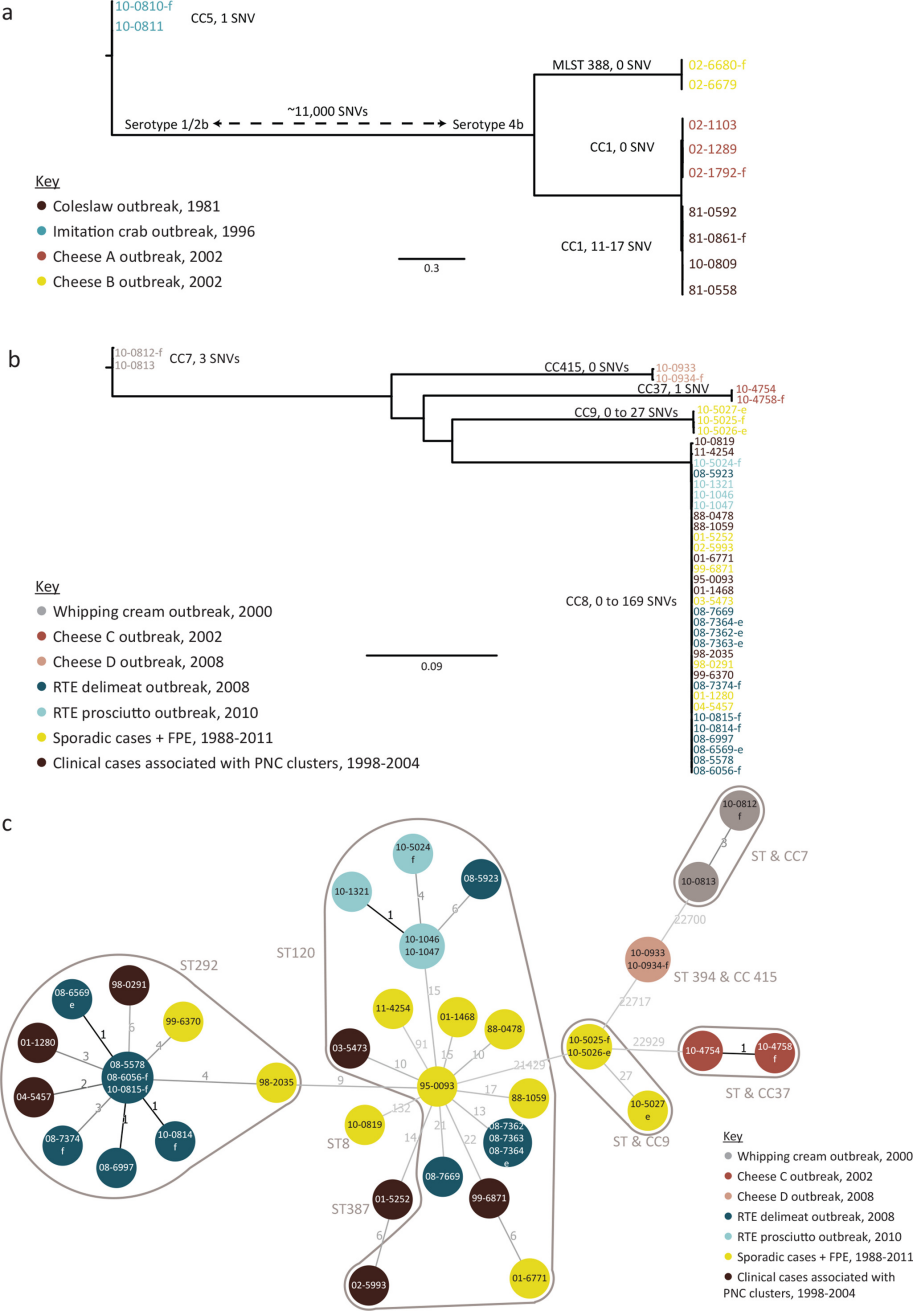
Shared hqSNV phylogenies for lineage I and lineage II *L. monocytogenes* isolates. Shared hqSNV phylogenies of (a) lineage I isolates (*n*=11, four outbreaks) maximum-likelihood tree based on 15 435 hqSNVs and a shared genome of 2 720 701 bp (reference: 81–0861, CP006874, 2 974 431 bp); and lineage II isolates (*n*=41, five outbreaks) maximum-likelihood (b) and minimum spanning (c) tree (MST) based on 49 359 hqSNVs and a shared genome of 2 709 893 bp (reference: 08–5578, CP001602 and CP001603); numbers and line weights on MST refer to the number of hqSNVs separating nodes. Scale bar represents nucleotide substitutions per site.

The shared genomes of *L. monocytogenes* lineages I and II were measured to be 2 720 701 bp (equivalent to 94.5 % of the reference genome 81–0861 and based on 11 isolates from four outbreaks) and 2 709893 bp [equivalent to 96.9 % of the reference genome 08–5578 and based on 41 isolates from five outbreaks, sporadic cases, and isolates recovered from the food processing environment (FPE) but not associated with disease], respectively. A larger amount of variation was observed within lineage II (53 859 SNVs or 1.98 % of the region compared) compared to lineage I where 0.61 % (16 364 SNVs) or more than a third less SNV variation was observed. These results are consistent with previous reports that lineage II encompasses a wider genetic diversity and that lineage I strains represent a highly clonal, homogeneous population, with reduced susceptibility to horizontal gene transfer and recombination compared to their lineage II cousins [6]. For all epidemiologically unrelated events studied, from either the same or differing MLST clonal complexes, the genomic variation acquired from both the species-specific and the lineage-specific shared genomes considerably improved genetic discrimination of isolates compared to conventional subtyping, revealing relationships congruent with epidemiological evidence (Figs 1, S2–S4; Tables S3–S6). However, increasing the size of the shared genome analysed to neither lineage-specific nor CC8-specific shared genetic content revealed significant additional hqSNV diversity among epidemiologically unrelated isolates (Table 1). In other words, the number of hqSNVs harvested, and thus the discriminatory power of hqSNV analysis, did not improve by increasing the size of the shared genome compared.

We speculate that the nearly consistent number of hqSNVs measured by all shared genome analytical approaches is a function of the relatively constant (as measureable) evolutionary rate of vertical inheritance targeted by the SNVPhyl approach and hence an approximately equal evolutionary rate for the entire collection of isolates studied. The genetic signature of vertical inheritance targeted probably represents the ‘true’ genetic core (i.e. highly stable, non-plastic regions of the genome), resulting in a highly accurate estimate of genetic relatedness, despite the amount of genome sampled. Additionally, the small handful of SNVs that distinguish outbreak-related isolates have a lower probability of being excluded from analysis as the shared genome sampled decreases; for instance, when reducing the analytical nucleotide set from 2.8 Mb to 2.6 Mb there is a low probability [(2.8–2.6)/2.8=7.1 %] of excluding the region containing a particular SNV. Thus the number of SNVs differing between two isolates from the same outbreak changes very little even though the region analysed, and the distance measurement, is determined by the input data sets. This relatively moderate effect of the input dataset on genetic distance (and therefore relatedness interpretations) is reassuring when faced with the non-standardized input datasets required for maximum genetic resolution and to support outbreak response decisions. Furthermore, SNVs between isolates belonging to a single outbreak are likely to be recently acquired, thus contributing to tail branch length and not divergence within the estimated phylogeny.

These observations are particularly relevant and timely to inform hqSNV and genomics-based MLST schemes for genomic epidemiology currently under development by public health and food safety agencies and international consortia [43, 44]. Large-scale sequencing projects for several bacterial pathogens are currently underway in Canada and globally to establish the role of shared genomes in the measurement of hqSNV distances and genetic relationships for recently diverged bacterial isolates.

### Population structure of clinical *L. monocytogenes*

Critical to the evaluation and comparison of bacterial subtyping schemes is an understanding of the overall diversity and prevalent clones for which the scheme is to be applied [20]. This large-scale survey to estimate the population structure of disease-causing *L. monocytogenes* in Canada was achieved by applying a well-defined and globally recognized MLST scheme for *L. monocytogenes* on a representative sampling of 536 retrospective clinical isolates recovered between 1981 and 2011 [10] (Fig. S5). During this period, 16 % of human listeriosis cases in Canada were caused by CC8, confirming the predominance of CC8 among listeriosis cases reported in prior smaller-scale studies [18, 19]. Whereas several MLST STs belonging to CC8 have been observed globally, in Canada CC8 isolates are predominantly observed as either ST 120 or ST 292 (Fig. S5). The resultant MLST datasets provided context for selection of outbreak-associated and sporadic isolates for our whole-genome characterizations (Fig. S5).

### Genetic relatedness revealed by WGS analyses correlate with conventional subtyping results

To assess the general concordance of traditional molecular typing schemes with whole-genome-based phylogenies, we performed phylogenetic analyses of 39 Canadian isolates combined with publicly available *L. monocytogenes* genomes using Mauve [45]. The resulting phylogenies placed the newly sequenced Canadian isolates as expected within the *L. monocytogenes* global phylogeny and validated the distribution of *L. monocytogenes* serotypes into three major phylogenetic clades according to evolutionary lineage as predicted by conventional serotyping (Tables S1, S7 and Fig. S6) [6, 10]. Although representing only a rudimentary estimate of phylogenetic relationships, this whole-genome dendrogram also supported relationships predicted by routine subtyping methods: PFGE (Fig. S7) and seven-gene MLST (Fig. S5), as well as relatedness determined by past traditional epidemiological investigations of outbreak events, except for isolates belonging to MLST CC8 (Table 1).

Switching to a reference-mapping approach and hqSNV analysis and reducing the comparison to include only *L. monocytogenes*-specific shared genetic content (2,790,598 bp representing 87.6 % of the reference genome 08–5578) did not improve discrimination of two CC8 outbreaks but continued to discriminate seven of nine studied outbreaks (Tables 1, S3 and Figs 1, S2) – comparable to PFGE and MLST which were also unable to distinguish these two CC8 outbreaks occurring 2 years apart (linked to RTE meats in 2008 and prosciutto in 2010). Next, reducing the genetic diversity of the input isolate set to only lineage II, and thus increasing the genetic content sampled to all sequences considered shared to lineage II strains (2 709 893 bp representing 97 % of the reference genome 08–5578), did not yield additional SNVs capable of distinguishing the two CC8 outbreaks based on the original case definitions (Fig. 1, Table S5).

**Fig. 2.**
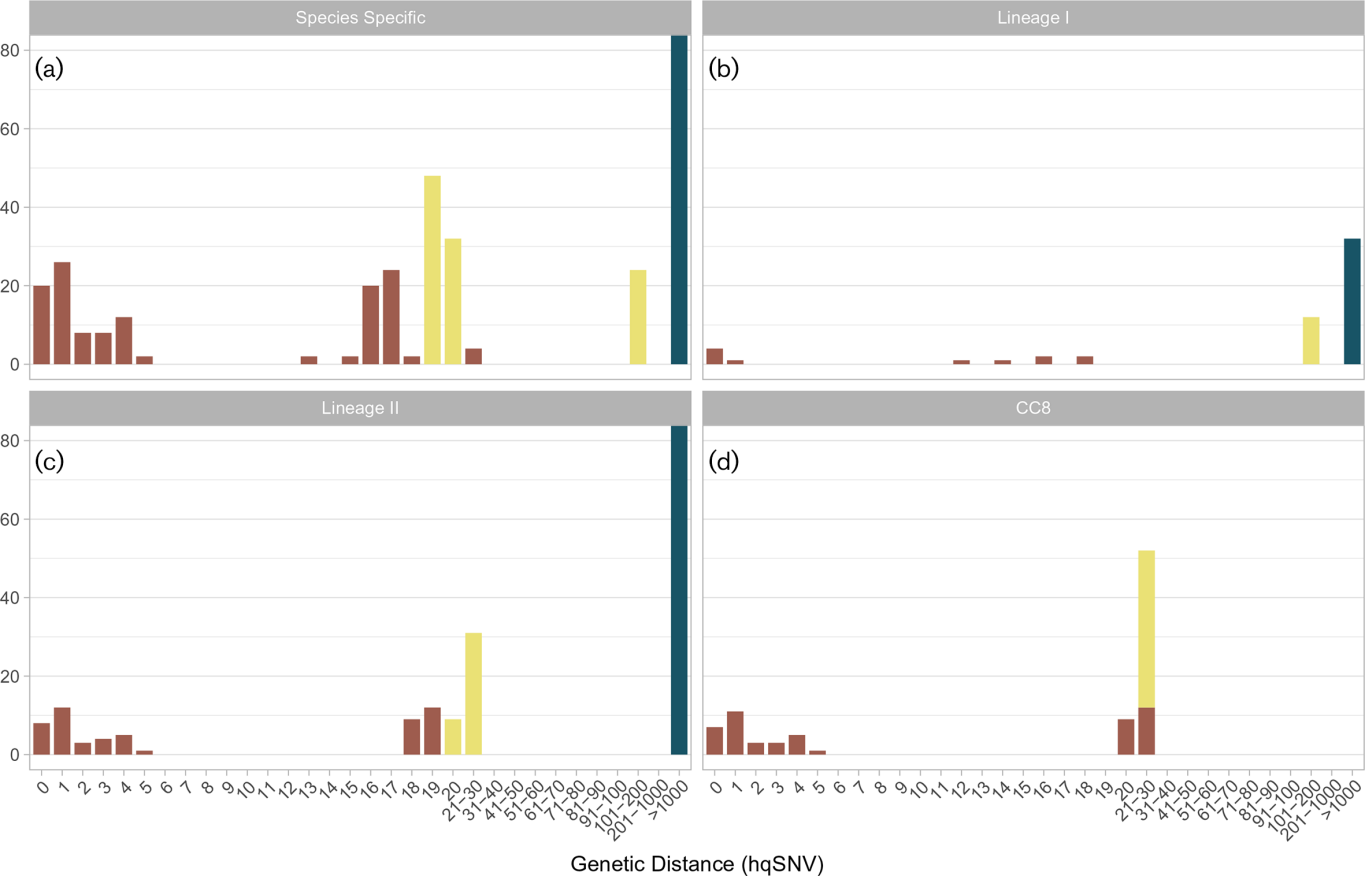
Isolate pair genetic distance analysis. The number of epidemiologically related isolate pairs (red), and pairs known to have no epidemiological link (yellow, same CC; blue, different CC) are plotted against their genetic distance (measured in hqSNVs) for the shared genomes of study isolates belonging to (a) *L. monocytogenes*; (b) lineage I; (c) lineage II; and (d) CC8.

To further resolve the two epidemiologically distinct CC8 outbreaks we compared isolates from these outbreaks against a diverse set of sporadic and cluster-related CC8 isolates from clinical cases representing a span of 24 years across Canada. This approach did not yield useful discriminatory information based on genetic distance alone (Fig. S4, Table S6). The pairwise genetic distances between isolates of known epidemiology were 0–35 and 0–5 hqSNVs for isolates belonging to the 2008 deli meat and 2010 prosciutto outbreaks, respectively. The pairwise genetic distance between the 2008 and 2010 outbreaks ranged from six to 34 hqSNVs and did not distinguish these CC8 outbreaks from each other. Expanding the input dataset to include a set of geographically and temporally diverse (presumably epidemiologically unrelated) sporadic isolates, the range of pairwise genetic distances of isolates with no known epidemiological relationship (*n*=412 pairs) was 2–183 hqSNVs (mean=37.5; median=24; mode=26) (Fig. 2, Tables S3, S5 and S6). Of these 412 pairs, 5.8 % of isolates were fewer than five hqSNVs apart. Similarly, 12.1 and 52.4 % of isolates were fewer than 10 and 25 hqSNVs apart, respectively. Notably the size of shared genome compared had little influence on the genetic distances observed between epidemiologically related pairs (Fig. 2). Consequently, the choice of reference sequence for read-based mapping approaches is more flexible than originally hypothesized.

**Fig. 3.**
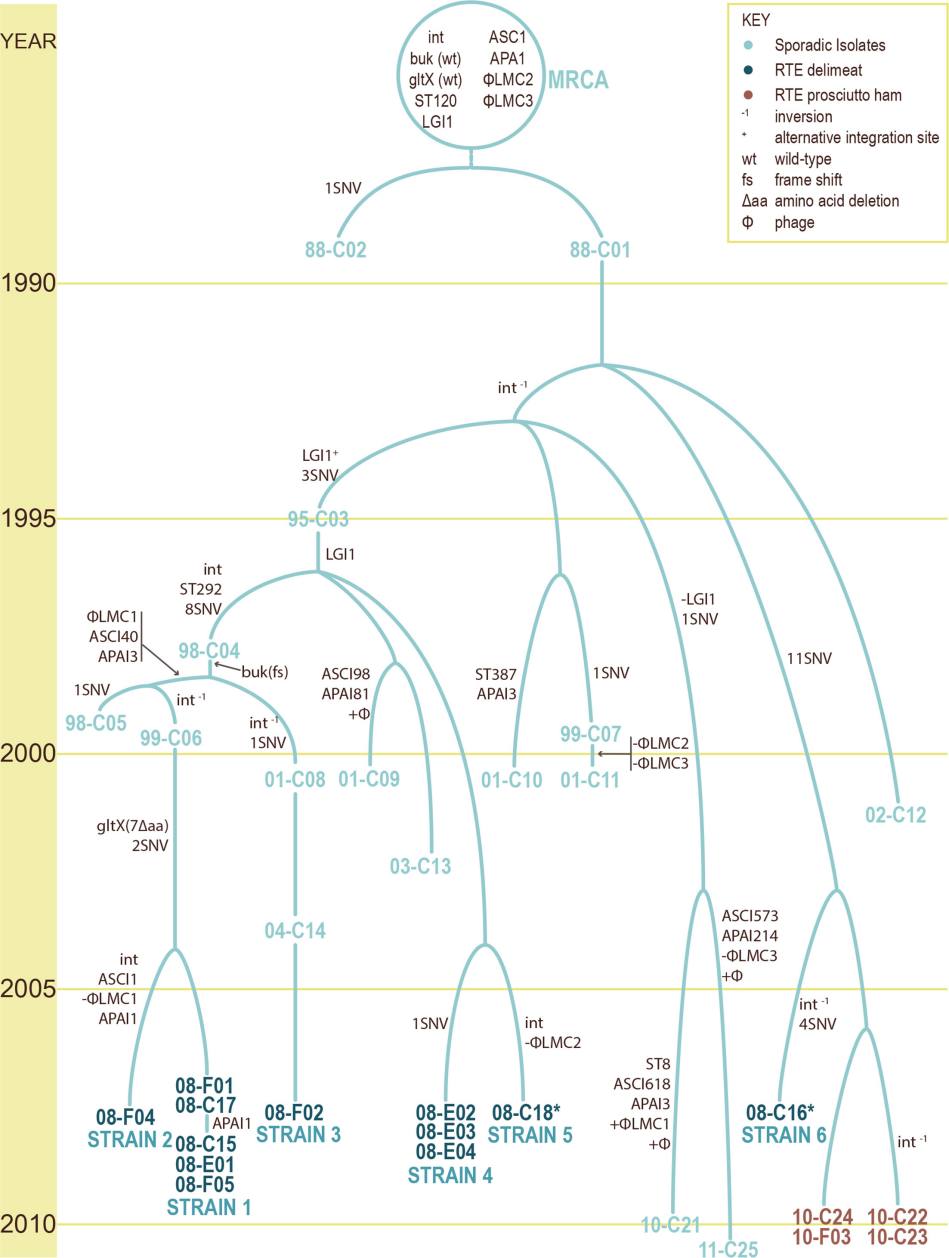
Chronophyletic model of CC8 evolution. Tree topology based on PCR-verified SNVs and macrodiverity for 32 CC8 *L. monocytogenes* isolates including two outbreaks and 15 sporadic cases. Time scale (left) and horizontal gene transfer events are indicated. PFGE (*AscI*) pattern and MLST classifications are listed for each isolate/strain. Branch labels show the number of canonical SNVs between isolates. Isolates from sporadic (light teal), 2010 prosciutto (brown) and 2008 deli meat (dark teal) investigations are indicated (inset). An asterisk (*) denotes cases that were probably not exposed to deli meat. CC8 strains described in Table 1 are indicated (bottom). MRCA, most recent common ancestor; *int*, integrase; *buk*, putative butyrate kinase; wt, wild type; *gltX*, putative glutamyl-tRNA synthetase; ϕLMC1/ϕLMC2/ϕLMC3, putative bacteriophage-related coding sequences; LGI1, listeria genomics island (50 kbp); ST, sequence type; aa, amino acid; fs, frame shift; Δ, deletion; ^−1^, inversion; ^+^, alternative integration site; SNV, single nucleotide variant; C, clinical isolate; F, food isolate; E, food processing equipment.

Many of these historical isolates have very close genetic distances (<5 hqSNVs) from isolates recovered across Canada and years apart (Fig. S4, Table S6). For example, two hqSNVs were observed between isolates recovered 4 years apart in different provinces (04–5457 vs 08–5578) (Table S1, Fig. S4). Unfortunately, the epidemiological evidence for these retrospective sporadic cases is unknown. It is possible that the Canadian CC8 *L. monocytogenes* clonality tests the limit of resolution even for WGS-based typing approaches to infer epidemiological relatedness (Fig. 3). Alternatively, genetically close CC8 isolates may share an un-investigated epidemiological relationship as was recently uncovered in an ice cream outbreak spanning 5 years [46]; or both may be linked to the same food establishment where listeria had colonized the processing facility over several years such as was observed by Orsi *et al.* [47].

This observation emphasizes that caution is needed to avoid inferring epidemiological relationships based on laboratory data alone. WGS is not the perfect subtyping tool negating the relevance of epidemiology. Epidemiological evidence is crucial and this premise remains unchanged from current and previous subtyping methods, wherein interpretation of results is performed using strict guidelines that must account for temporal and other epidemiological contexts [20, 48, 49]. For example, we know that FPEs can be colonized by *L. monocytogenes* over decades, causing sporadic cases that may go unlinked to a source and that the resulting *L. monocytogenes* food and clinical isolates may acquire as few as one confirmed canonical SNV in their entire shared genome over a span of 12 years [47]. Yet the expected genetic distance between epidemiologically related isolates remains to be defined, and the CC8 outbreak examples explored in this study illustrate the challenge of distinguishing isolates even at maximum genetic resolution offered by closed finished genome evolutionary modelling.

### Indistinguishable PFGE patterns are susceptible to subtype reversions and cannot be assumed monophyletic

To elucidate why shared genome analyses could not distinguish two temporally and epidemiologically distinct CC8 outbreaks, we expanded our comparison to include all genome content in an evolutionary model containing historically recovered clinical CC8 isolates to inform divergence of the CC8 clone (Fig. 3). This CC8 chronophyletic evolutionary model, based on the full complement of evolutionary changes identified among all CC8 isolates sampled – from SNVs, multinucleotide variants (MNVs) and indels, to horizontally transferred elements – accounts for the strain delineations observed by the whole genome comparisons, and shared genome hqSNV analyses.

This model infers that all CC8 isolates are presumed to have descended from a most recent common ancestor (MRCA), harbouring a specific set of genetic traits. This encompasses wild-type alleles of *gltX* and *buk* that were identified in a previous comparison of 08–5578 and 08–5923 and used to generate the initial 2008 CC8 outbreak evolutionary model [7]: presence of LGI1, φLMC2 and φLMC3, a PFGE *Asc*I macrorestriction pattern LMACI.0001, and MLST ST120. This ancestor is also presumed to have harboured a single indel: a phage integrase carried within the genome of 08–5578 but absent in 08–5923.

Fig. 3 demonstrates the parsimony-inferred evolutionary relationship between CC8 isolates and reveals several interesting linkages. Firstly, the 2010 prosciutto outbreak isolates share a close genetic relationship to each other, and are well delineated from the other CC8 isolates. As shown in Fig. 3, the MRCA initially branches into two lineages based on a single canonical SNV between two sporadic clinical isolates from 1988 (88–1059 and 88–0478). All other CC8 isolates analysed in our study are presumed to share a recent ancestor with 88–0478, descending via one of two ancestral lineages that emerged between 1988 to 1995 and was driven by SNV diversification and phage acquisition and loss.

Conventional epidemiological evidence also indicates that PFGE subtype reversions have obscured the true genetic relationship between PFGE-matching isolates belonging to CC8. A predicted evolutionary relationship of CC8 isolates from two outbreaks, seven PulseNet Canadaclusters and six sporadic cases indicates that associated PFGE patterns, LMACI.0001 and LMACI.0040, are not monophyletic (Fig. 3). PFGE pattern reversions have occurred during CC8 evolution due to prophage acquisition and loss, obscuring the true phylogenetic relationship between CC8 isolates/strains, and this challenges the interpretation of laboratory evidence during public health/food safety investigations. Thus caution must be applied when including horizontally acquired elements to infer phylogenetic relateness.

The primary restriction enzyme, *Asc*I, yielded two variant patterns among isolates recovered from the implicated products and FPE [5]. In the 2008–2010 genomics investigation of the 2008 RTE deli meat outbreak, Gilmour *et al.* [7] demonstrated that φLMC1 integration into the tRNA-Ser gene in 08–5578 accounts for the observed shift in *Asc*I restriction pattern (from LMACI.0040 to LMACI.0001) between 08–5578 and 08–5923; otherwise these isolates were genetically highly similar at the SNV level (in 2008 estimated to be roughly 28 SNVs apart) [7]. These key findings and the available evidence in 2008 supported the outbreak response to include both LMACI.0040 and LMACI.0001 in the clinical outbreak case definition. The majority of clinical cases yielded isolates with LMACI.0040 while only a few clinical cases had LMACI.0001 [5]. Exposure to the implicated product was not documented for three of four patients from whom LMACI.0001 isolates were recovered, but these clinical cases were included in the investigation based on the recovery of contemporary LMACI.0001 isolates directly from the implicated products and FPE. A PFGE-*Apa*I variant was also observed in the investigation; however, as *Apa*I is the secondary enzyme and the *Apa*I results did not alter interpretation of the outbreak scenario, the *Apa*I variant was not investigated further.

Phylogenetic analysis of CC8 based on varying genetic content and differing methodologies yielded the same results: at least six distinct strains were associated with the 2008 outbreak investigation, but it appears that a single strain caused all clinical cases associated with the contaminated product (Table 1, Fig. 3). The main 2008 outbreak strain ‘Strain 1’ is represented by all LMACI.0040 clinical case isolates sampled along with some PFGE-matching RTE meat isolates; all ‘Strain 1’ isolates shared the molecular subtypes LMACI.0040 and seven-gene MLST ST292. Three additional variant strains were recovered from the implicated product and/or the FPE (all were LMACI.0001 and ST292/ST120), but by all analyses presented in this study, the LMACI.0001 isolates recovered from the implicated food and FPE in 2008 are not genetically related to the LMACI.0001 clinical isolates associated with the 2008 outbreak (designated ‘Strain 5’, represented by isolate 08–7669, and ‘Strain 6’, represented by isolate 08–5923). Maximum-parsimony analyses (goeBURST), presented as MSTs, provide additional visualization of the studied CC8 datasets and further evidence to support the strain delineations observed by each level of shared genome analysis (Figs 1c, 3 and S4). Taken together, this new evidence does not correlate with the laboratory evidence in 2008 and challenges a reinterpretation of the epidemiological evidence.

All levels of WGS analyses supported the close genetic relationship of ‘Strains 1 to 4’ (the main 2008 outbreak strain and isolates recovered from the implicated product and FPE) and could be considered a single strain with an allowable variation of 0–4 hqSNVs (Table 1). The chronophyletic analysis in this study suggests that these strains have been coevolving in the food processing facility since the 1990s – alternatively, a common source has repeatedly reintroduced strains 1–4 and/or their common ancestors over the last two decades (Fig. 3). The approaches presented in this study use genetic distance measured in SNVs, and attempt to identify a distance threshold of relatedness for defining WGS clusters for public health interpretation and response. However, the CC8 outbreak challenge described in this study warrants investigation in supplementing the SNV distance parameter with clear delineations for (1) SNVs indicating divergence (canonical SNVs) and (2) SNVs that contribute to branch length within a clade. Such SNV distinctions could improve the discriminatory power and epidemiological concordance for investigations of clusters or outbreaks caused by highly clonal bacterial pathogens.

Taken together, these results clearly demonstrate that indistinguishable PFGE patterns (or even highly related genomes) alone do not unequivocally support that an epidemiological relationship exists, an already well-established limitation of foodborne molecular epidemiology [20, 48] and further inform the replacement of conventional subtyping with WGS-based evidence to support outbreak response decisions.

### MLVA had the lowest discriminatory power of all subtyping methods applied

In an attempt to genetically distinguish two otherwise separate CC8 outbreaks we surveyed an altogether different genetic target, VNTRs, measured by capillary electrophoresis as repeat regions are known to be problematic to WGS acquisition and analysis. MLVA for foodborne pathogens such as shiga-toxin producing *E. coli* (STEC) is a highly discriminatory subtyping method, with strain resolution comparable to PFGE and possibly WGS (http://www.pulsenetinternational.org/protocols/mlva/). The MLVA scheme for *L. monocytogenes*, however, was not able to distinguish between CC8 outbreaks, nor between isolates belonging to the same CC. Six isolates belonging to CC1, from two distinct outbreaks 21 years apart, shared identical MLVA profiles (Table 1). Similarly, 16 isolates belonging to CC8 from two distinct outbreaks 2 years apart shared identical MLVA profiles; furthermore, all CC8 isolates tested dating back to 1988 shared identical MLVA profiles with the exception of a single 2002 clinical isolate differing from the main CC8 MLVA profile by a single repeat at a single locus.

### Reinterpretation of the 2008 epidemiological evidence supports genetic relationships identified by WGS

The inability of each of the applied methods to distinguish two CC8 outbreaks is based solely on isolates from two clinical cases (08–5923 and 08–7669). These isolates exhibited PFGE pattern LMACI.0001, a single band variant of the main outbreak pattern LMACI.0040 (Fig. S7). Only one of four cases exhibiting isolates with the LMACI.0001 subtype ate/probably ate the implicated product, compared to 50 of 54 (93 %) of cases exhibiting isolates with the main outbreak pattern LMACI.0040 [5]; however, inclusion of the variant PFGE cases into the outbreak case definition during the 2008 investigation was based on established guidelines, during a simultaneous public health crisis caused by a strain of highly similar PFGE pattern. Additionally, this was the first application of WGS to a real-time public health investigation and while a single prophage and approximately 28 SNVs differentiated the PFGE variant strain from the main outbreak strain, at that time, there was no previous public health experience or scientific understanding of what differences to expect between inclusion/exclusion outbreak isolates [7, 20].

For the purposes of this retrospective study, removing the two clinical isolates without solid food exposure evidence from this outbreak allowed resolution of all nine outbreaks by seven-gene MLST and all levels of hqSNV analyses. The case of two nearly indistinguishable outbreaks presented in this study underlines the critical role that epidemiology plays in the interpretation of laboratory evidence. When interpreted together, genetic and epidemiological evidence create a powerful synergy to infectious disease surveillance and control.

### Single strain definition in the WGS era is complex and subjective

Foodborne disease laboratory surveillance systems rely on the initial assumption that an outbreak is caused by a single strain (as defined by conventional subtyping) resulting from a single contamination event and brief exposure, leading to the observation of indistinguishable or highly similar ‘variant’ subtypes that are more likely to have originated from a common point source and ancestor compared to strains with differing subtypes [48, 50]. However, in defining the ‘outbreak strain’, outbreak investigators are often challenged with a background of sporadic cases of varying relatedness to the ‘outbreak strain’ and a barrage of often highly genetically related strains recovered from suspected foods and FPEs. We have naively expected WGS to resolve all of our highly clonal, problematic subtypes but this study highlights that that may not always be the case – even at maximum genetic resolution.

Overall, the absolute genetic distances observed in this study between fully closed and finished genomes of epidemiologically related food and clinical *L. monocytogenes* isolates support this default single strain theory of foodborne disease investigation for eight of nine outbreaks in this study (Table 1; Fig. 3). For four of nine outbreaks, the related food and clinical isolates were 100 % identical across their entire closed finished genomes; and for two additional outbreaks, the full extent of genetic difference between the closed finished genomes of the related food and clinical isolates was three SNVs or less (Table 1). The 2008 and 2010 outbreaks attributed to CC8, after a close scrutiny of CC8 micro- and macro-genetic differences and re-review of epidemiological data, also revealed very close genetic distances between the clinical and food isolates, differing by ≤2 and ≤5 hqSNVs in their shared genomes, respectively (Table 1). Additional strains represented by food and FPE isolates recovered during the 2008 investigation had a genetic distance from the clinical strain no greater than 21 shared genome hqSNVs. In only one of the nine outbreaks studied was the number of hqSNVs between the clinical isolate(s) and attributed food isolate(s) greater than three hqSNVs (Fig. 2); the 1981 outbreak revealed a genetic distance of 11–18 hqSNVs amongst the shared genomes of three clinical isolates and a single coleslaw isolate. The sole food isolate (81–0861) associated with this significant outbreak has a variant but highly similar PFGE-*Asc*I pattern; however, this preliminary WGS analysis of the 1981 outbreak challenges how we define the limits of acceptable variation for a single strain/WGS match in the NGS era.

### WGS evidence may generate epidemiological hypotheses about source

Considering the membership of the coleslaw outbreak strain(s) to a globally recognized clonal complex (CC1) it was surprising to observe a relatively large amount of hqSNV variation compared to the other eight outbreaks in this study, which each contained less than three confirmed SNVs across their entire finished genomes (Table 1). In addition to underlying genetic diversity among the 1981 coleslaw outbreak isolates, this outbreak differs from the other eight outbreaks studied by the source of contamination. One could argue that the age and storage conditions of the 1981 isolates contributed to the diversity observed; however, subsequent sequencing of all outbreak isolates, including genetically identical fetal–maternal isolate pairs, showed this not to be the case (data not shown). The food, cabbage, was almost certainly contaminated with *L. monocytogenes* at the cabbage field, a natural and inherently microbially diverse environment. Weller *et al.* [51, 52] found that irrigation with ground or rainwater yielded point source contamination events but that agricultural samples were contaminated with higher microbial diversity after treatment with manure and that the diversity effect persisted even years after manure was applied. Thus, the application of manure to the cabbage field could have led to an outbreak with more diversity than would be expected for a point source event. Furthermore, lineage I strains are more often associated with agricultural soil and cattle watersheds compared with other *L. monocytogenes* lineages [6].

By comparison, the remaining eight outbreaks in this study were most likely contaminated at the food production level (industrial production facility or private kitchen) which would, by comparison, be expected to harbour a smaller, less diverse population than the natural soil environment of a cabbage field and thereby result in a more clonal outbreak. The 2011 cantaloupe outbreak in the United States is another example where contamination that probably began in the field or during shipping yielded gross food contamination and clinical cases with an enormous range of genetic diversity, including several strains from both lineages I and II [53, 54]. Notably, however, two of the cheese outbreaks studied (attributed to cheeses A and D, in 2002 and 2008, respectively) were associated with aged raw milk cheese. Thus, *L. monocytogenes* could have entered the cheese via milk contaminated at the bovine/farm level although most RTE foods are contaminated post-processing [4, 5, 15]. Only two isolates were sequenced for each of the raw milk cheese outbreaks with none and three confirmed SNVs across their entire finished genomes. Expanded studies are required to confirm the ‘contamination scope – SNV diversity correlation’ hypothesis.

Resolving multi-strain events is an ongoing public health challenge and is reliant on good epidemiological data compared to more typical point source, single strain outbreaks. However, if confirmed, the ‘contamination scale – SNV diversity correlation’ hypothesis could be useful during a temporal or variant subtype cluster investigation, where the underlying diversity within a cluster of disease cases may help focus the epidemiological investigation towards possible source types (i.e. foods contaminated in the natural environment such as produce, fruit and vegetables – cantaloupe and cabbage; or food contaminated in a production facility environment – processed meats and cheeses).

Knowledge of gene content at the whole genome level has the potential to assist public health and food safety investigators to (1) generate source hypotheses during outbreak investigations and (2) provide useful information about source attribution. For example, the 2008 deli-meat outbreak strains each contained a genomic island coding for tolerance to quaternary ammonium compounds, common sanitizers used in food processing facilities [55]. Future genomic studies of gene content may reveal additional biomarkers that hint at a strain’s host and/or ecological environmental history and help to guide public health response activities [50, 56].

### WGS sequencing can significantly assist foodborne outbreak investigations involving multiple variant subtypes wherein the epidemiology is inherently more challenging

The genetic distances between epidemiologically related food and clinical *L. monocytogenes* isolates, measured at the whole genome, *L. monocytogenes* shared and lineage-specific shared levels indicated a very close genetic relationship (0–18 hqSNVs) for 75 isolate pairs belonging to the same outbreak despite PFGE and MLST variation observed in some outbreaks (Table 1). The genetic distances measured between outbreak-related isolate pairs are significantly less than genetic distances measured between 390 isolate pairs known to have no epidemiological relationship and belonging to different MLST CCs (4256–74370 hqSNVs). However, distinct outbreaks belonging to the same CC may be as close as 7–9 hqSNVs apart, such as for the 2008 and 2010 CC8 outbreaks, or may be separated by as much as 91–169 hqSNVs, such as for two CC1 outbreaks, 21 years apart. It should also be noted that while epidemiologically unrelated isolates belonging to the same CC may appear to be closely genetically related by hqSNV analyses, further assessment of the differences in the accessory genomes of isolates during public health and food safety investigations could readily provide the discriminatory resolution necessary to genetically distinguish strains within CCs [43] (as shown in Fig. 3). For example, mobile genetic elements acquired prior to the contamination/outbreak event and vertically inherited by isolates associated with the same epidemiological event could also yield clues useful for genomic epidemiology investigations. *L. monocytogenes* isolates that share elements of accessory genome are more likely to share genetic ancestry [57] and this may be exploited in cases where the resolving power of vertically inherited SNVs is limited, such as the Canadian CC8 story described in this study. Thus, implementation of WGS for routine surveillance may provide substantial improvements to both sensitivity and specificity of outbreak response and allow hypotheses about outbreak strain history or success based on specific genetic content that is often horizontally acquired [50].

The genetic distance values measured in this study for *L. monocytogenes* are consistent with values measured for other foodborne bacterial pathogens such as *E. coli* with a common source threshold estimated to be less than or equal to five SNVs [24], and common clonal serovars of *Salmonella* Enteritidis (0–1 hqSNV) [23], among others [58].

Genetic distances, measured in shared genome hqSNVs extracted from WGS data, are a useful biological indicator to estimate genetic relatedness that may show excellent congruence with epidemiological relatedness. Thus, the analytical approach and hqSNV distance values measured in this study will support the development of PulseNet-specific interpretative criteria for *L. monocytogenes* WGS cluster detection and outbreak response during the next generation of food safety and public health investigations. Critically, as with all subtyping methods preceding WGS analyses, conclusions on epidemiological relatedness can only be achieved collectively, and on a case-by-case basis with careful consideration of all available information. For example, future investigations may further challenge SNV distance as the most accurate marker to imply epidemiological relationships.

Moving forward, large-scale retrospective and prospective WGS studies, driven nationally by PulseNet Canada and internationally by public health and food safety agencies and consortia (P. Gerner-Smidt and S. Brisse, personal communications [59]), are in progress to populate public health databases and provide continued refinement of WGS markers and interpretive criteria for *L. monocytogenes* and other bacterial pathogens, providing imperative confidence in WGS results for responsible food safety and public health application. This study provides the scientific community with a unique dataset (2010, 2014, and closed finished qualities) with which to test WGS-schema alternatives to hqSNV analyses, such as ‘whole genome MLST’ approaches based on the *L. monocytogenes* shared or pangenomes [60, 61]. These latter approaches offer potential resolution comparable to hqSNV analysis by capturing single-base mutations while moderating the effects of horizontal gene transfer and homologous recombination events through allele-based cluster analysis, and more importantly for real-time public health surveillance, they facilitate rapid data sharing for inter-laboratory communication of WGS subtype results. The high-quality datasets generated by this study provide a means of method calibration needed to interpret WGS data for responsible public health and food industry decision-making.

### Conclusions

Harnessing the synergistic power of epidemiological and genomic data is the next revolution in the public health management of infectious disease. The critical role of epidemiological data interpretation is highlighted by the CC8 story outlined in this study – in such cases of highly clonal organism groups, harnessing the power of that synergy requires flexible yet robust informatics tools capable of high-quality phylogenetic inferences [58, 62]. Integration of analysis tools that combine high-volume laboratory and epidemiological data will allow for unprecedented evidence generation supporting endemic and outbreak disease investigation.

Overall, the comparative analyses presented in this study demonstrate that listeriosis outbreaks are typically monophyletically distinct events with very few SNVs present between isolates from the same event, although an unusually high level of SNV diversity within an event might be due to the microbial diversity inherent to the source of contamination (e.g. soil) and/or the investigative environment sampled. Furthermore, standard molecular subtyping methods, PFGE, MLST and MLVA, predict relationships that are highly concordant with those predicted by WGS and NGS analyses in most cases; however, in the case of a highly prevalent PFGE/MLST clone, e.g. CC8, indistinguishable PFGE patterns are not monophyletic, resulting from repeated acquisition and loss of prophages that obscure genetic relationships estimated by vertical inheritance trait acquisition. In these cases WGS analysis is able to provide both higher resolution and higher epidemiological concordance. Moreover, we have clearly demonstrated a role for WGS, and specifically hqSNV analyses based on vertical inheritance, in informing an outbreak event by providing genetic evidence to support the epidemiological data in the 2008 deli meat outbreak. Although strains with different MLST and PFGE types belonging to the same MLST CC were recovered, the SNV-variant-based and macrodiversity analysis revealed that a single strain led to the outbreak, and that three additional highly related strains were recovered during the food safety investigation. Furthermore, expanded analysis of CC8 isolates recovered over a 30-year time span has revealed genetic events underlying the evolution of this clone and identified SNV-level changes as the main driver of clonal evolution leading to the emergence of a clone with the capacity to cause large-scale foodborne outbreaks [43].

The sequences generated and phylogenetic analyses presented in this study will provide the base evidence for interpretive criteria and guidelines for WGS typing to support routine public health and food safety surveillance and response activities. Furthermore, the bioinformatics tools [22, 63] developed in support of, and parallel to, this study provide a means of robust phylogenetic analyses in support of public health and food safety activities.

## Data bibliography

1. The draft and finished genomes are available www.ncbi.nlm.nih.gov under BioProjects PRJNA167872 to PRJNA167908, PRJNA215182 to PRJNA215193, PRJNA36361 and PRJNA36363 (please refer to Table S1 for isolate-specific accession numbers).

## Supplementary Data

Supplementary File 1
