# Supplementary material for: Shared genome analyses of notable listeriosis outbreaks, highlighting the critical importance of epidemiological evidence, input datasets and interpretation criteria
Source: Microb Genom. 2019 Jan 16;5(1):e000237. doi: 10.1099/mgen.0.000237 (PMC6412057; doi:10.1099/mgen.0.000237)
Supplement: Supplementary File 1 [file mgen-5-237-s001.pdf]

SUPPLEMENTAL DATA

Figure S1. Robustness of reference-based shared genome hqSNV analysis pipeline. Maximum likelihood phylogenetic analyses for 39 *L. monocytogenes* isolates using data collected in (A) 2010 using GAIIx/454/Sanger and (B) 2014 using high quality MiSeq data (136,302 and 142,935 shared hqSNVs, respectively) (refer to **Methods**). Technical sequencing replicates generated by different platforms, laboratories/users, sample preparation methods, and with various data qualities and read lengths exhibit similar phylogenetic topologies and genetic distances. Genetic distance bars indicating the shared hqSNV frequency are shown. Reference genome: 08-5578 (3,032,624 bp); shared genome sizes: 2,554,801 bp (2010 data set) and 2,596,205 bp (2014 data set).

Figure S2. Species-wide *L. monocytogenes* shared genome hqSNV phylogeny. Species-wide shared hqSNV phylogeny for *L. monocytogenes* isolates (*n*=52, 9 distinct outbreaks). A total of 143,045 hqSNV positions were identified across a shared genome of 2,595,673 bp. Reference genome: 08-5578. Additional details in **Methods**. (A) Minimum spanning tree, Isolates are colour-coded by epidemiology (inset). MLST clonal complex (CC) groups and number of hqSNVs between isolates are indicated. Food isolate, f; food processing equipment isolate, e; ready-to-eat, RTE. (B) Maximum likelihood tree.

Figure S3. *L. monocytogenes* Lineage I shared hqSNV phylogeny. Shared hqSNV phylogeny for lineage I *L. monocytogenes* isolates (*n*=11, 4 distinct outbreaks). A total of 15,435 hqSNV positions were identified across a shared genome of 2,720,701 bp. Reference genome: 81-0861. Additional details in **Methods**. (A) Minimum spanning tree; Isolates are colour-coded by epidemiology (inset); MLST clonal complex (CC) groups and number of hqSNVs between isolates are indicated; Food isolate, f; food processing equipment isolate, e; ready-to-eat, RTE. (B) Maximum likelihood tree.

Figure S4. *L. monocytogenes* CC8 shared hqSNV phylogeny. Shared hqSNV phylogeny for Clonal Complex 8 (CC8) lineage II *L. monocytogenes* isolates (*n*=32, 2 distinct outbreaks and 15 sporadic cases) (**Table S1**). A total of 361 hqSNV positions were identified across a shared genome of 2,790,598 bp. Reference genome: 08-5578. (A) Minimum spanning tree (B) Maximum likelihood tree. Isolates are colour-coded by epidemiology (inset). MLST clonal complex (CC) groups and number of hqSNVs between isolates are indicated. Food isolate, f; food processing equipment isolate, e; ready-to-eat, RTE.

Figure S5. Population structure of clinical *Listeria monocytogenes* in Canada. The population structure of 536 disease-causing *L. monocytogenes* from Canada (1981-2013) represented by a 7-gene MLST-based minimum-spanning tree generated in BioNumerics v6.5 [1]. Nodes correspond to sequence type (ST); node diameters are proportional the frequency of each ST, and the serotype is indicated (colour legend). Branch numbers show the number of allelic differences between connected STs. Grey zones identify STs in the same clonal complex (CC).

Figure S6. Whole-genome Comparison of Canadian Isolates With Publicly Available *L. monocytogenes* Genomes. Neighbour-joining phylogeny of 39 finished *L. monocytogenes*

Canadian genomes (**Table S1**) and 23 publically available genomes (**Table S7**) based on global pairwise alignments generated using ClustalW [2] and MAUVE v20150226 [3]. Epidemiologically related isolates listed in are highlighted, and serotypes and lineages are shown. The blue zone indicated CC8 isolates. A genetic distance bar (bottom left) is indicated.

Figure S7. PFGE analysis of *L. monocytogene*s isolates in this study. PFGE dendrograms for 42 *L. monocytogenes* isolates were generated using standard PulseNet protocols and BioNumerics v6.5 and *AscI* (**Panel A**) or *ApaI* (**Panel B**) restriction enzymes. The PFGE pattern identification and *p*-values for dendrogram nodes are shown. NLEP#, isolate name. Additional details in **Methods**.

Table S1. Canadian *Listeria monocytogenes* isolates analyzed in this study.

Table S2A. Shared hqSNV matrix for 39 *L. monocytogenes* isolates sequenced in 2010. A total of 136,302 hqSNV positions were identified across a shared genome of 2,554,801 bp. Reference genome: 08-5578. Additional details in **Methods**.

Table S2B. Shared hqSNV matrix for 39 *L. monocytogenes* isolates sequenced in 2013. A total of 142,935 hqSNV positions were identified across a shared genome of 2,596,205 bp. Reference genome: 08-5578. Additional details in **Methods**.

Table S3. Species-wide shared hqSNV matrix for *L. monocytogenes* isolates (*n*=52, 9 distinct outbreaks). A total of 143,045 hqSNV positions were identified across a shared genome of 2,595,673 bp. Reference genome: 08-5578. Additional details in **Methods**.

Table S4. Shared hqSNV matrix for lineage I *L. monocytogenes* isolates (*n*=11, 4 distinct outbreaks). A total of 15,435 hqSNV positions were identified across a shared genome of 2,720,701 bp. Reference genome: 81-0861. Additional details in **Methods**.

Table S5. Shared hqSNV matrix for lineage II *L. monocytogenes* isolates (*n*=41, 5 distinct outbreaks). A total of 49,359 hqSNV positions were identified across a shared genome of 2,709,893 bp. Reference genome: 08-5578. Additional details in **Methods**.

Table S6. Shared hqSNV matrix for Clonal Complex 8 (CC8) lineage II *L. monocytogenes* isolates (*n*=32, 2 distinct outbreaks and 15 sporadic cases) (**Table S1**). A total of 361 hqSNV positions were identified across a shared genome of 2,790,598 bp. Reference genome: 08-5578. Additional details in **Methods**.

Table S7. Publically available *Listeria monocytogenes* genomes used in this study.

Table S8. Sequences of substituted primers used for MLST.

A

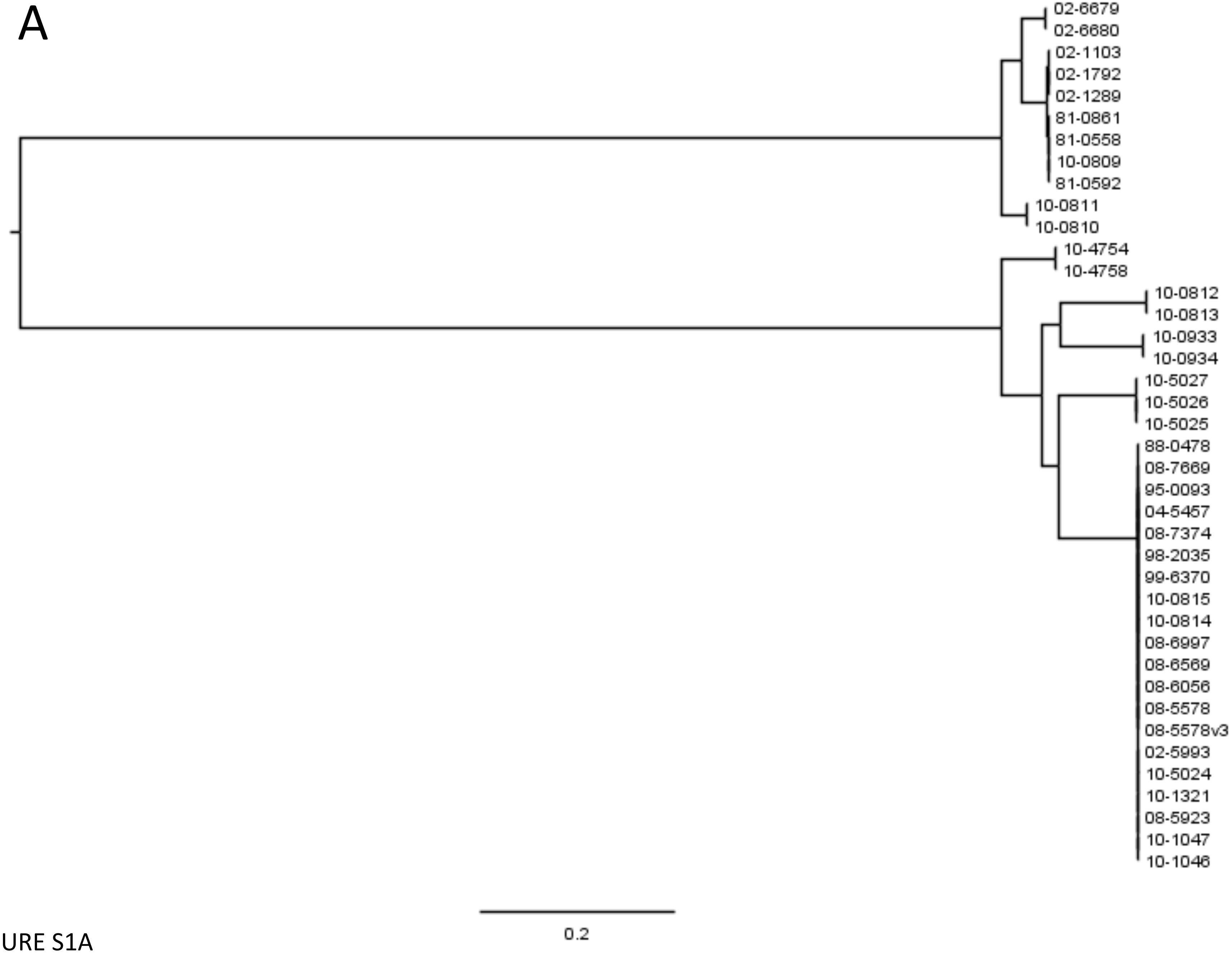
02-6679
02-6680
02-1103
02-1792
02-1289
81-0861
81-0558
10-0809
81-0592
10-0811
10-0810
10-4754
10-4758
10-0812
10-0813
10-0933
10-0934
10-5027
10-5026
10-5025
88-0478
08-7669
95-0093
04-5457
08-7374
98-2035
99-6370
10-0815
10-0814
08-6997
08-6569
08-6056
08-5578
08-5578v3
02-5993
10-5024
10-1321
08-5923
10-1047
10-1046
0.2

FIGURE S1A

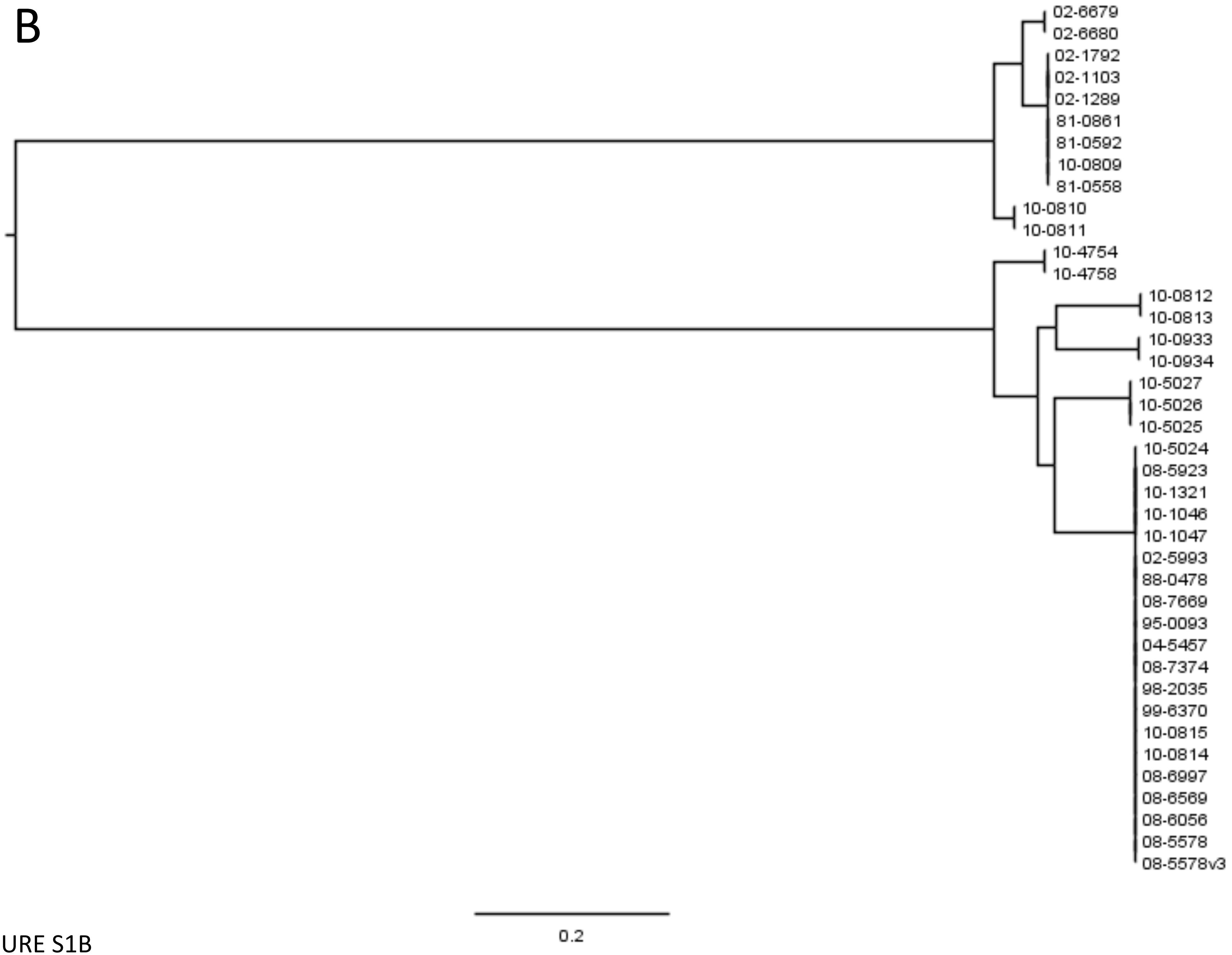
B
02-6679
02-6680
02-1792
02-1103
02-1289
81-0861
81-0592
10-0809
81-0558
10-0810
10-0811
10-4754
10-4758
10-0812
10-0813
10-0933
10-0934
10-5027
10-5026
10-5025
10-5024
08-5923
10-1321
10-1046
10-1047
02-5993
88-0478
08-7669
95-0093
04-5457
08-7374
98-2035
99-6370
10-0815
10-0814
08-6997
08-6569
08-6056
08-5578
08-5578v3
0.2

FIGURE S1B

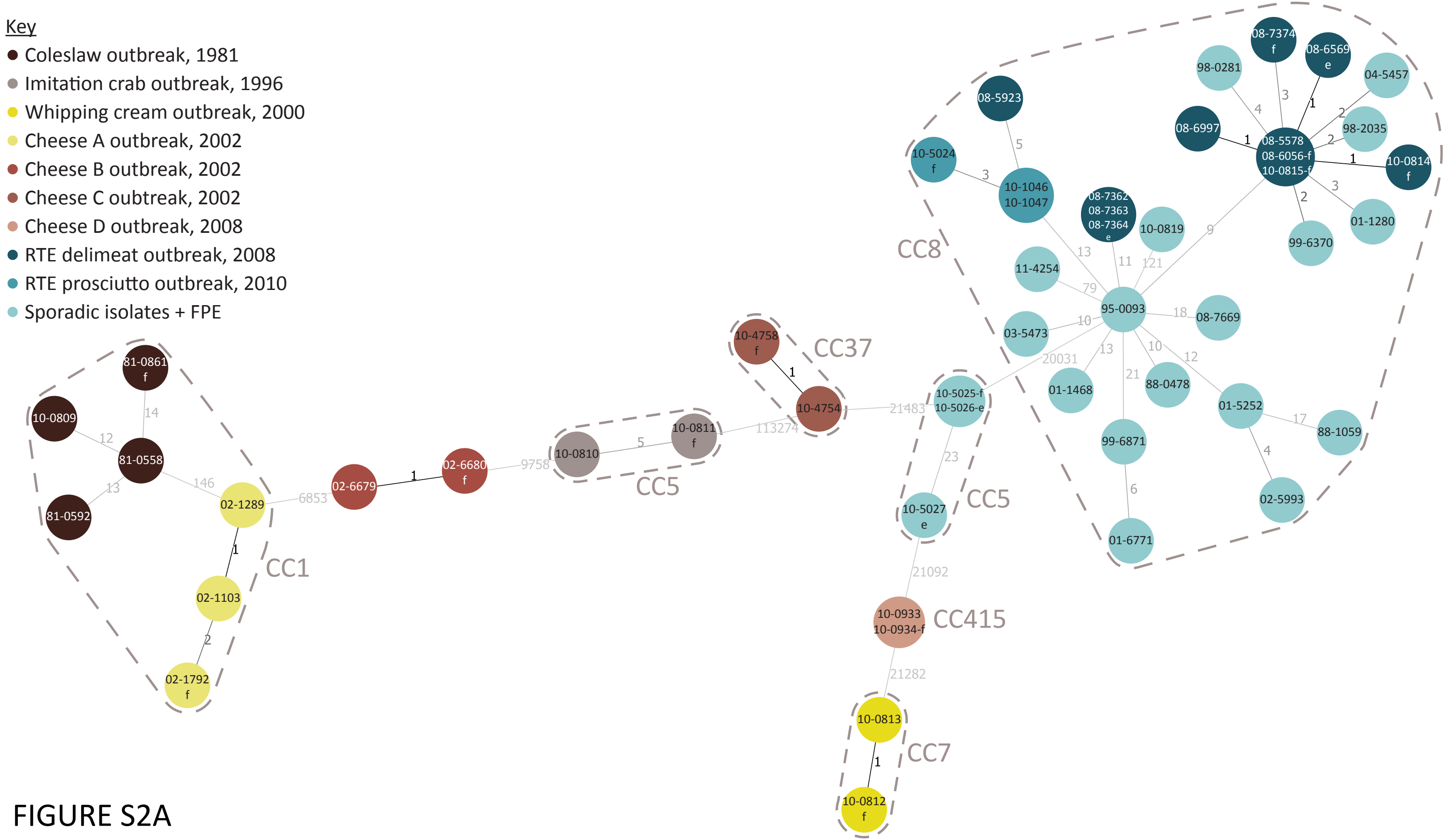
Key
Coleslaw outbreak, 1981
Imitation crab outbreak, 1996
Whipping cream outbreak, 2000
Cheese A outbreak, 2002
Cheese B outbreak, 2002
Cheese C oubtreak, 2002
Cheese D outbreak, 2008
RTE delimeat outbreak, 2008
RTE prosciutto outbreak, 2010
Sporadic isolates + FPE
CC1
81-0861 f
10-0809
81-0558
81-0592
02-1289
02-1103
02-1792 f
02-6679
02-6680 f
10-0810
10-0811 f
CC5
10-4758 f
10-4754
CC37
10-5025-f 10-5026-e
10-5027 e
CC5
10-0933 10-0934-f
CC415
10-0813
10-0812 f
CC7
CC8
95-0093
08-5578 08-6056-f 10-0815-f
08-7374 f
08-6569 e
98-0281
04-5457
98-2035
08-6997
10-0814 f
01-1280
99-6370
08-5923
10-5024 f
10-1046 10-1047
08-7362 08-7363 08-7364 e
10-0819
11-4254
03-5473
08-7669
01-1468
88-0478
01-5252
88-1059
99-6871
02-5993
01-6771

FIGURE S2A

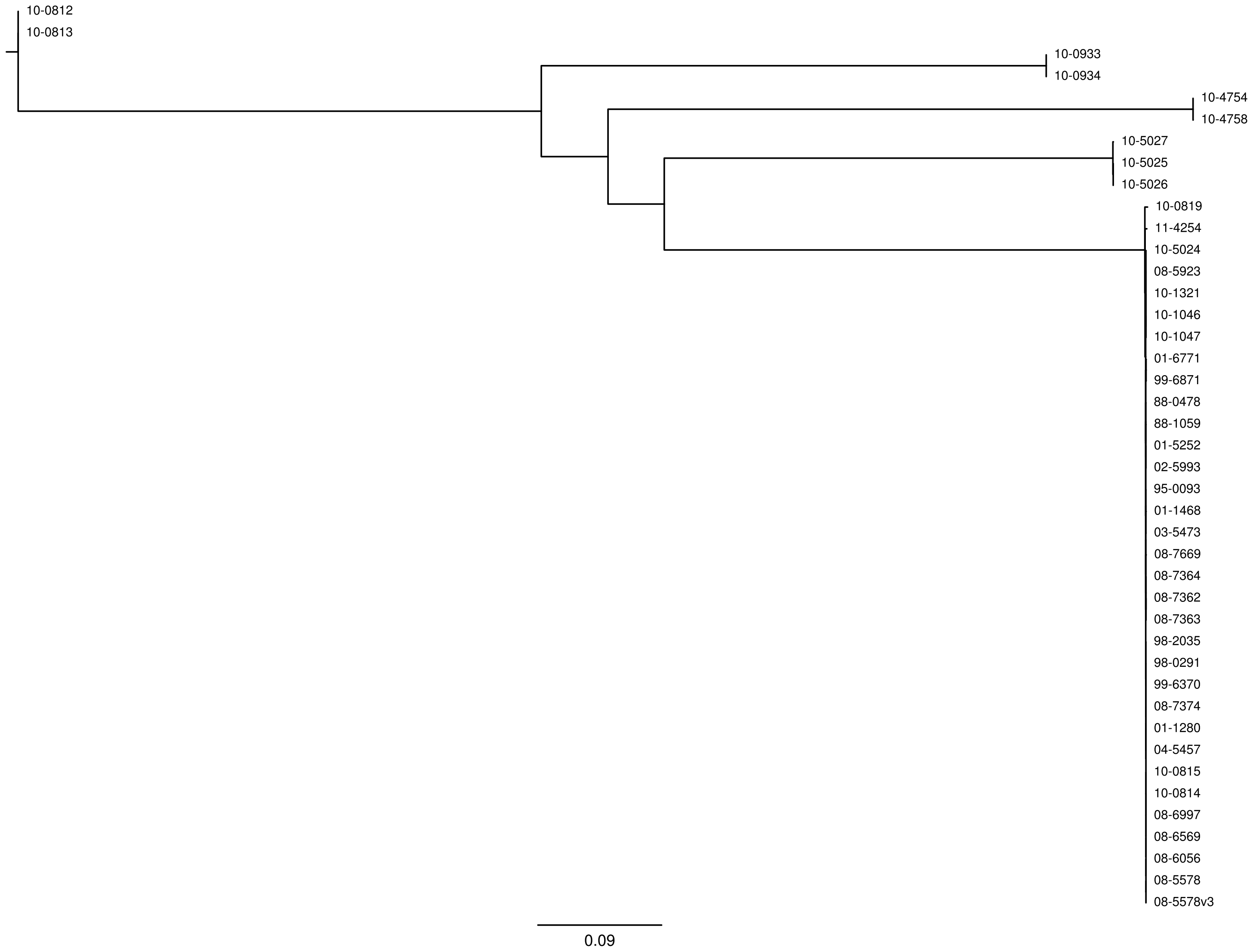
10-0812
10-0813
10-0933
10-0934
10-4754
10-4758
10-5027
10-5025
10-5026
10-0819
11-4254
10-5024
08-5923
10-1321
10-1046
10-1047
01-6771
99-6871
88-0478
88-1059
01-5252
02-5993
95-0093
01-1468
03-5473
08-7669
08-7364
08-7362
08-7363
98-2035
98-0291
99-6370
08-7374
01-1280
04-5457
10-0815
10-0814
08-6997
08-6569
08-6056
08-5578
08-5578v3
0.09

FIGURE S2B

FIGURE S3

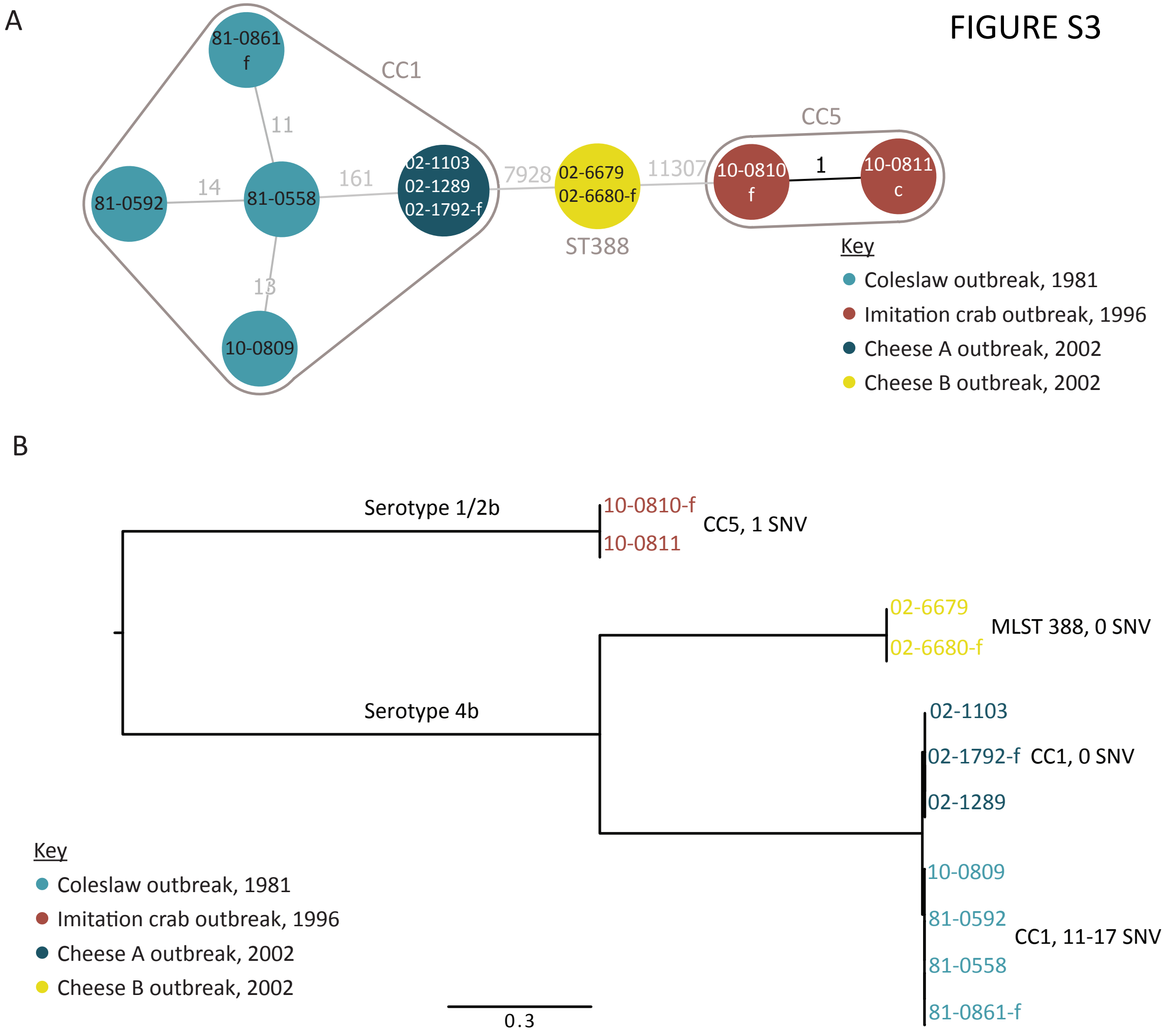
A
CC1
81-0861
f
11
81-0592
14
81-0558
161
02-1103
02-1289
02-1792-f
13
10-0809
7928
02-6679
02-6680-f
ST388
11307
CC5
10-0810
f
1
10-0811
c
Key
Coleslaw outbreak, 1981
Imitation crab outbreak, 1996
Cheese A outbreak, 2002
Cheese B outbreak, 2002
B
Serotype 1/2b
10-0810-f
10-0811
CC5, 1 SNV
02-6679
02-6680-f
MLST 388, 0 SNV
Serotype 4b
02-1103
02-1792-f
CC1, 0 SNV
02-1289
10-0809
81-0592
CC1, 11-17 SNV
81-0558
81-0861-f
Key
Coleslaw outbreak, 1981
Imitation crab outbreak, 1996
Cheese A outbreak, 2002
Cheese B outbreak, 2002
0.3

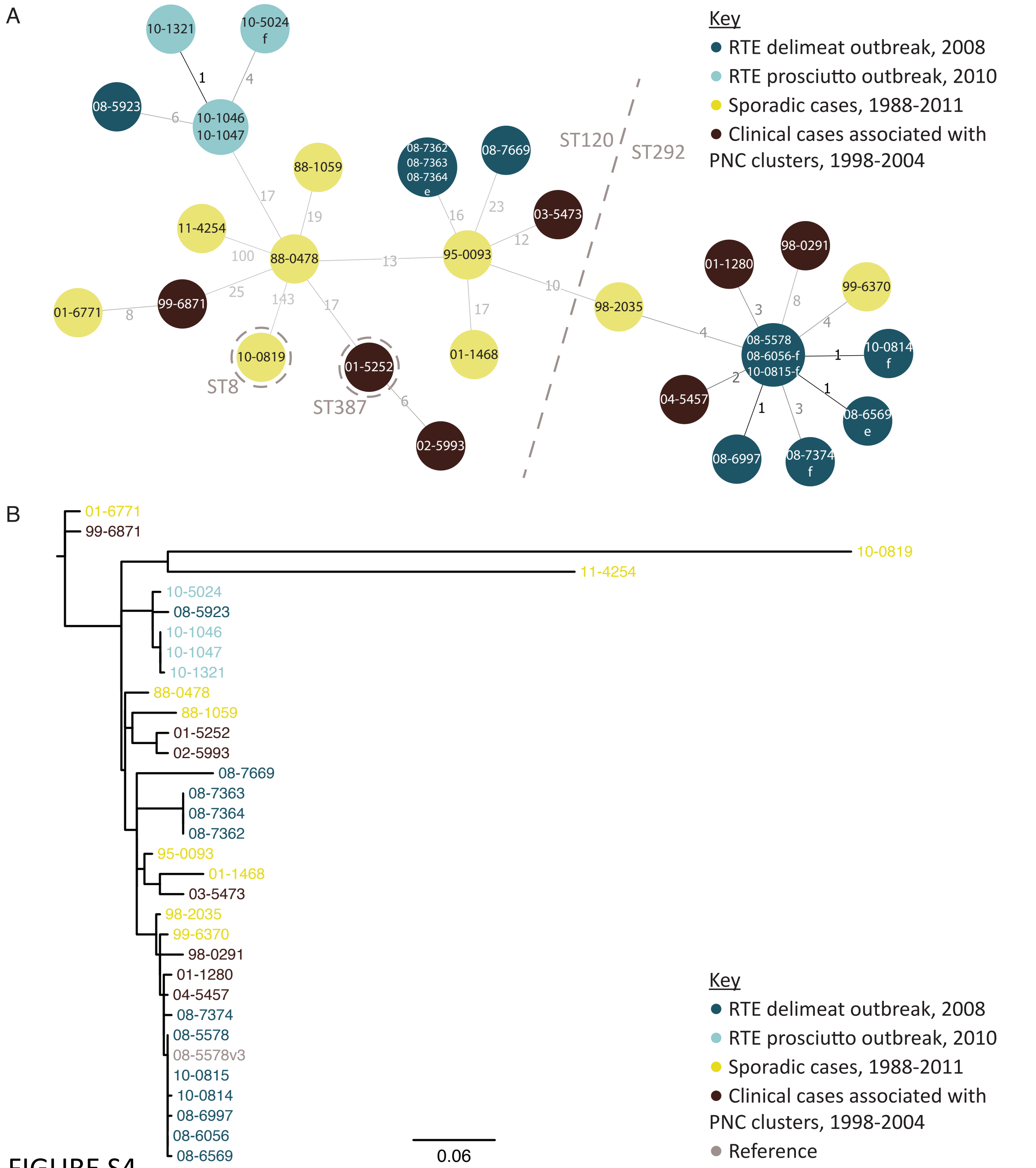
A
Key
RTE delimeat outbreak, 2008
RTE prosciutto outbreak, 2010
Sporadic cases, 1988-2011
Clinical cases associated with PNC clusters, 1998-2004
ST120
ST292
ST8
ST387
10-1321
10-5024 f
08-5923
10-1046 10-1047
88-1059
11-4254
88-0478
01-6771
99-6871
10-0819
01-5252
02-5993
08-7362 08-7363 08-7364 e
08-7669
95-0093
03-5473
01-1468
98-2035
01-1280
98-0291
99-6370
08-5578 08-6056-f 10-0815-f
10-0814 f
08-6569 e
08-7374 f
08-6997
04-5457
B
01-6771
99-6871
10-0819
11-4254
10-5024
08-5923
10-1046
10-1047
10-1321
88-0478
88-1059
01-5252
02-5993
08-7669
08-7363
08-7364
08-7362
95-0093
01-1468
03-5473
98-2035
99-6370
98-0291
01-1280
04-5457
08-7374
08-5578
08-5578v3
10-0815
10-0814
08-6997
08-6056
08-6569
0.06
Key
RTE delimeat outbreak, 2008
RTE prosciutto outbreak, 2010
Sporadic cases, 1988-2011
Clinical cases associated with PNC clusters, 1998-2004
Reference

FIGURE S4

Multi-Locus Sequence Typing (MLST) Minimum Spanning Tree (MST) Canadian *L. monocytogenes* Isolates, all isolates in database as of 2013-06

MLST method by Ragon et al. 2008 with alignment and MST created via Bionumerics v6.5; numbers on branches indicate allele differences between connected sequence types (ST); node diameter is proportional to number of isolates; node colour shows isolate serotype.

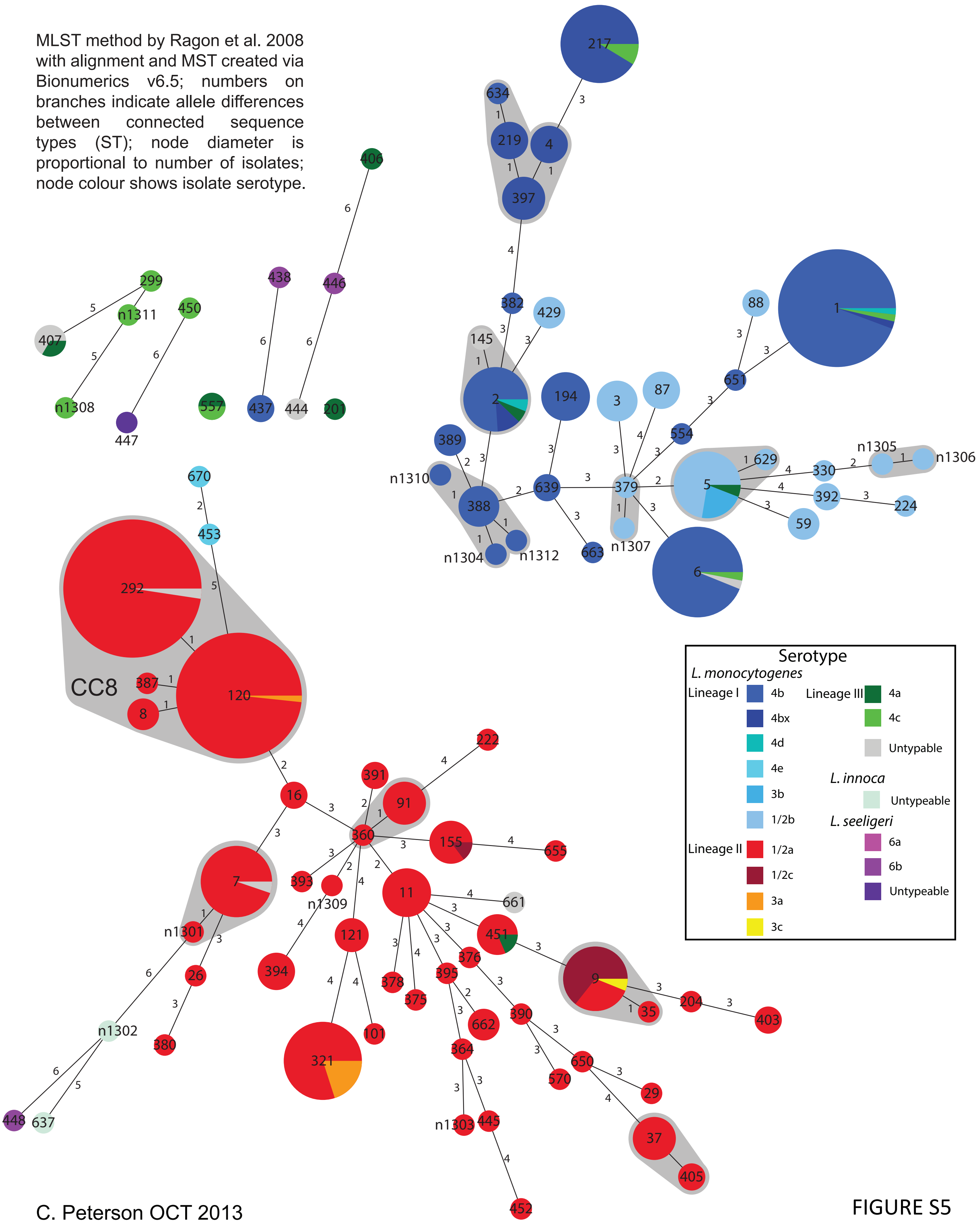

C. Peterson OCT 2013

FIGURE S5

FIGURE S6

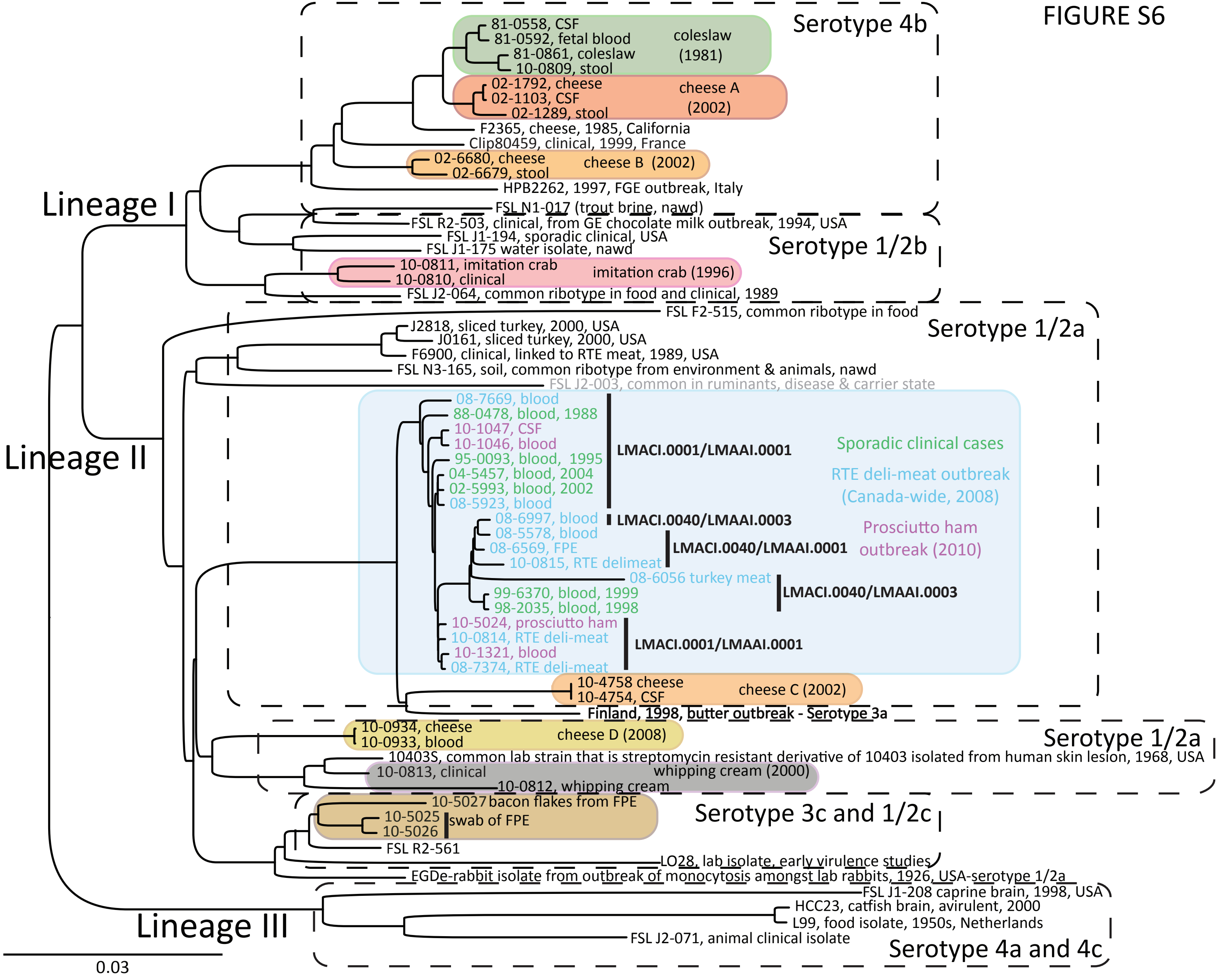
Serotype 4b
81-0558, CSF
81-0592, fetal blood
81-0861, coleslaw
10-0809, stool
coleslaw (1981)
02-1792, cheese
02-1103, CSF
02-1289, stool
cheese A (2002)
F2365, cheese, 1985, California
Clip80459, clinical, 1999, France
02-6680, cheese
02-6679, stool
cheese B (2002)
HPB2262, 1997, FGE outbreak, Italy
Lineage I
FSL N1-017 (trout brine, nawd)
FSL R2-503, clinical, from GE chocolate milk outbreak, 1994, USA
FSL J1-194, sporadic clinical, USA
FSL J1-175 water isolate, nawd
Serotype 1/2b
10-0811, imitation crab
10-0810, clinical
imitation crab (1996)
FSL J2-064, common ribotype in food and clinical, 1989
FSL F2-515, common ribotype in food
J2818, sliced turkey, 2000, USA
J0161, sliced turkey, 2000, USA
F6900, clinical, linked to RTE meat, 1989, USA
FSL N3-165, soil, common ribotype from environment & animals, nawd
FSL J2-003, common in ruminants, disease & carrier state
Serotype 1/2a
08-7669, blood
88-0478, blood, 1988
10-1047, CSF
10-1046, blood
95-0093, blood, 1995
04-5457, blood, 2004
02-5993, blood, 2002
08-5923, blood
LMACI.0001/LMAAI.0001
Sporadic clinical cases
RTE deli-meat outbreak (Canada-wide, 2008)
08-6997, blood
08-5578, blood
08-6569, FPE
10-0815, RTE delimeat
LMACI.0040/LMAAI.0003
LMACI.0040/LMAAI.0001
Prosciutto ham outbreak (2010)
08-6056 turkey meat
99-6370, blood, 1999
98-2035, blood, 1998
LMACI.0040/LMAAI.0003
10-5024, prosciutto ham
10-0814, RTE deli-meat
10-1321, blood
08-7374, RTE deli-meat
LMACI.0001/LMAAI.0001
Lineage II
10-4758 cheese
10-4754, CSF
cheese C (2002)
Finland, 1998, butter outbreak - Serotype 3a
10-0934, cheese
10-0933, blood
cheese D (2008)
Serotype 1/2a
10403S, common lab strain that is streptomycin resistant derivative of 10403 isolated from human skin lesion, 1968, USA
10-0813, clinical
whipping cream (2000)
10-0812, whipping cream
10-5027 bacon flakes from FPE
10-5025
10-5026
swab of FPE
Serotype 3c and 1/2c
FSL R2-561
LO28, lab isolate, early virulence studies
EGDe-rabbit isolate from outbreak of monocytosis amongst lab rabbits, 1926, USA-serotype 1/2a
FSL J1-208 caprine brain, 1998, USA
HCC23, catfish brain, avirulent, 2000
L99, food isolate, 1950s, Netherlands
FSL J2-071, animal clinical isolate
Lineage III
Serotype 4a and 4c
0.03

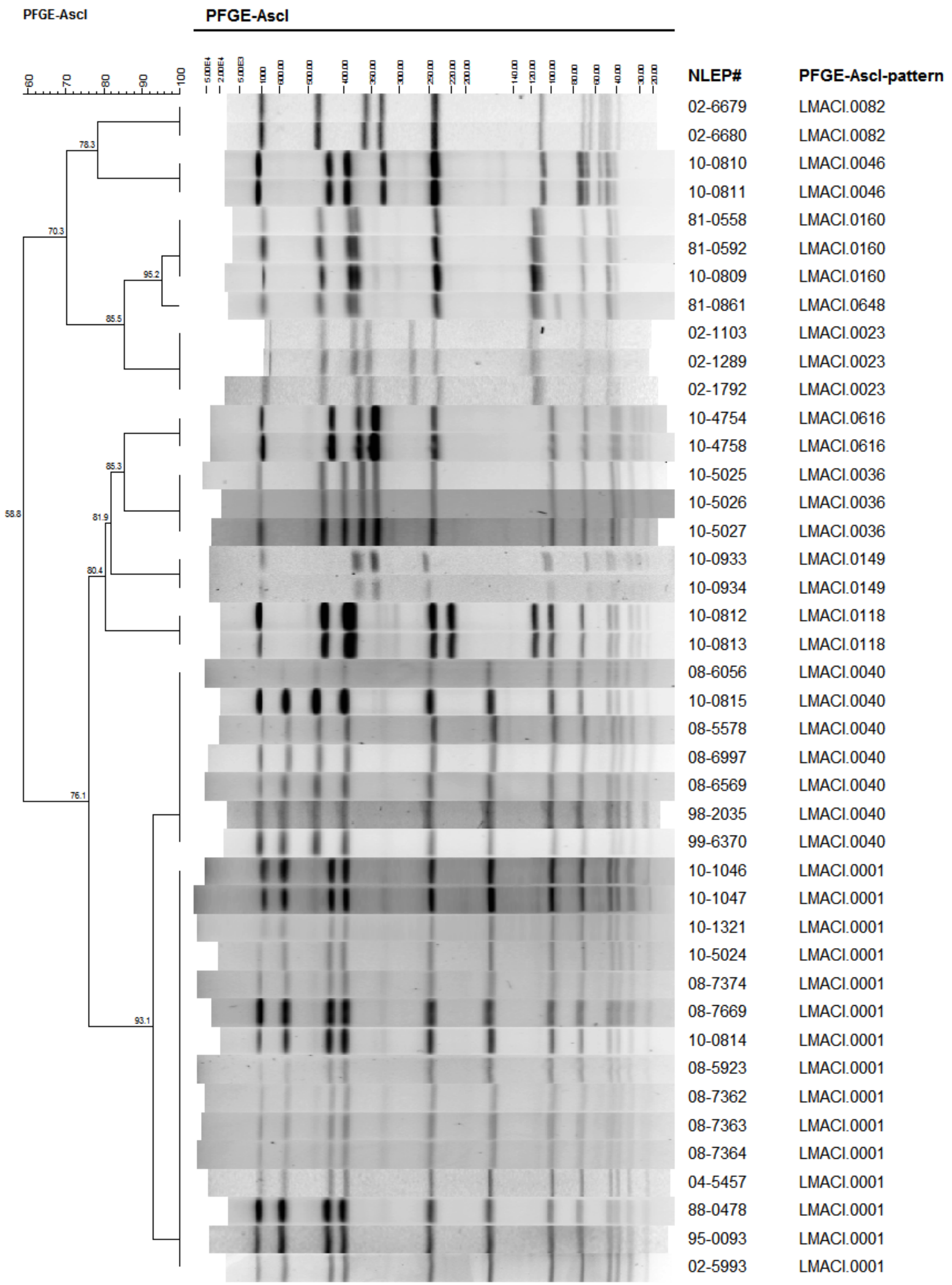
PFGE-AscI
PFGE-AscI
60 70 80 90 100
78.3
70.3
95.2
85.5
85.3
81.9
80.4
58.8
76.1
93.1
NLEP# PFGE-AscI-pattern
02-6679 LMACI.0082
02-6680 LMACI.0082
10-0810 LMACI.0046
10-0811 LMACI.0046
81-0558 LMACI.0160
81-0592 LMACI.0160
10-0809 LMACI.0160
81-0861 LMACI.0648
02-1103 LMACI.0023
02-1289 LMACI.0023
02-1792 LMACI.0023
10-4754 LMACI.0616
10-4758 LMACI.0616
10-5025 LMACI.0036
10-5026 LMACI.0036
10-5027 LMACI.0036
10-0933 LMACI.0149
10-0934 LMACI.0149
10-0812 LMACI.0118
10-0813 LMACI.0118
08-6056 LMACI.0040
10-0815 LMACI.0040
08-5578 LMACI.0040
08-6997 LMACI.0040
08-6569 LMACI.0040
98-2035 LMACI.0040
99-6370 LMACI.0040
10-1046 LMACI.0001
10-1047 LMACI.0001
10-1321 LMACI.0001
10-5024 LMACI.0001
08-7374 LMACI.0001
08-7669 LMACI.0001
10-0814 LMACI.0001
08-5923 LMACI.0001
08-7362 LMACI.0001
08-7363 LMACI.0001
08-7364 LMACI.0001
04-5457 LMACI.0001
88-0478 LMACI.0001
95-0093 LMACI.0001
02-5993 LMACI.0001

FIGURE S7A

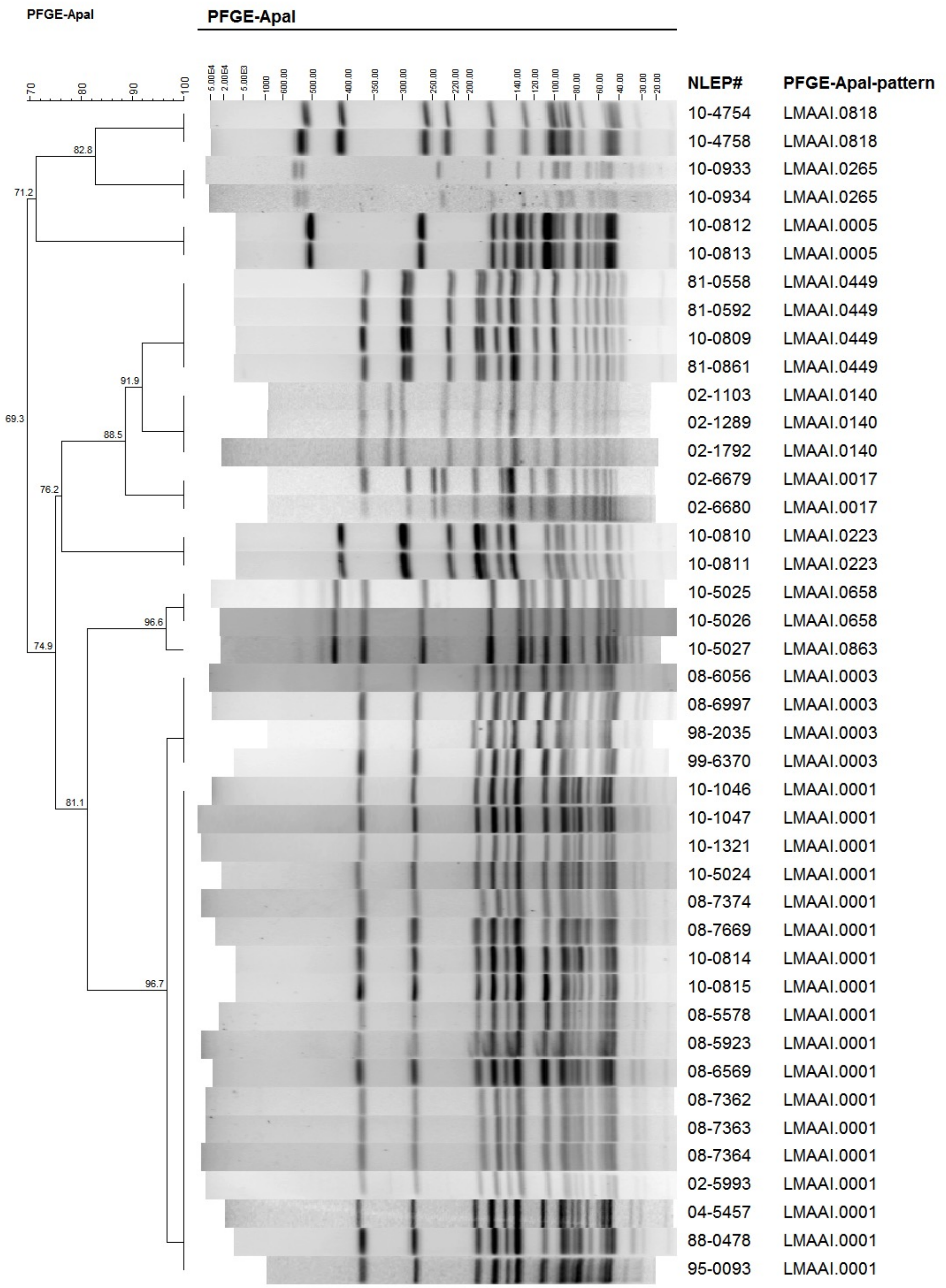
PFGE-ApaI
PFGE-ApaI
70
80
90
100
82.8
71.2
69.3
91.9
88.5
76.2
96.6
74.9
81.1
96.7
NLEP#
PFGE-ApaI-pattern
10-4754 LMAAI.0818
10-4758 LMAAI.0818
10-0933 LMAAI.0265
10-0934 LMAAI.0265
10-0812 LMAAI.0005
10-0813 LMAAI.0005
81-0558 LMAAI.0449
81-0592 LMAAI.0449
10-0809 LMAAI.0449
81-0861 LMAAI.0449
02-1103 LMAAI.0140
02-1289 LMAAI.0140
02-1792 LMAAI.0140
02-6679 LMAAI.0017
02-6680 LMAAI.0017
10-0810 LMAAI.0223
10-0811 LMAAI.0223
10-5025 LMAAI.0658
10-5026 LMAAI.0658
10-5027 LMAAI.0863
08-6056 LMAAI.0003
08-6997 LMAAI.0003
98-2035 LMAAI.0003
99-6370 LMAAI.0003
10-1046 LMAAI.0001
10-1047 LMAAI.0001
10-1321 LMAAI.0001
10-5024 LMAAI.0001
08-7374 LMAAI.0001
08-7669 LMAAI.0001
10-0814 LMAAI.0001
10-0815 LMAAI.0001
08-5578 LMAAI.0001
08-5923 LMAAI.0001
08-6569 LMAAI.0001
08-7362 LMAAI.0001
08-7363 LMAAI.0001
08-7364 LMAAI.0001
02-5993 LMAAI.0001
04-5457 LMAAI.0001
88-0478 LMAAI.0001
95-0093 LMAAI.0001

FIGURE S7B

Table S1. Canadian Listeria monocytogenes isolates analyzed in this study

Outbreak Investigation	Isolate	Figure 3 designation	Year Isolated	Submitting Province/Region	Specimen Type	Source	Serotype	MLST	Clonal complex	PFGE *AscI*	PFGE *ApaI*	Epidemiology	Reference	Assembly status	Genome Accession	Bioproject	BioSample
Coleslaw, 1981	81-0861	NA	1981	Nova Scotia	Food	Coleslaw	4b	1	1	LMACI.0648	LMAAI.0449	41 cases; 17 deaths including 2 adults with meningitis, 4 stillbirths, 5 perinatal	[19]	Complete	CP006874	PRJNA167874	SAMN05771116
	10-0809	NA	1981	Nova Scotia	Clinical	Stool	4b	1	1	LMACI.0160	LMAAI.0449			Complete	CP007167	PRJNA167875	SAMN07124730
	81-0592	NA	1981	Nova Scotia	Clinical	Fetal blood	4b	1	1	LMACI.0160	LMAAI.0449			Complete	CP007526	PRJNA167873	SAMN02713778
	81-0558	NA	1981	Nova Scotia	Clinical	CSF	4b	1	1	LMACI.0160	LMAAI.0449			Complete	CP007525	PRJNA167872	SAMN02713777
Imitation crab, 1996	10-0811	NA	1996	Ontario	Food	Imitation crab	1/2b	5	5	LMACI.0046	LMAAI.0223	2 cases: 0 deaths, severe gastroenteritis, 1 septic and hospitalized	Farber JM, Daley EM, MacKie MT, Limerick B: A small outbreak of listeriosis potentially linked to the consumption of imitation crab meat. Lett Appl Microbiol 2000, 31(2):100-104.	Complete	CP007169	PRJNA167877	SAMN07124732
	10-0810	NA	1996	Ontario	Clinical	Stool	1/2b	5	5	LMACI.0046	LMAAI.0223			Complete	CP007168	PRJNA167876	SAMN07124731
Whipping cream, 2000	10-0812	NA	2000	Manitoba	Food	Whipping Cream	1/2a	7	7	LMACI.0118	LMAAI.0122	7 cases: 6 mild, 1 hospitalized and CSF positive	Pagotto F, Ng LK, Clark C, Farber J, Canadian Public Health Laboratory Network: Canadian listeriosis reference service. Foodborne Pathog Dis 2006, 3(1):132-137.	Complete	CP007170	PRJNA167878	SAMN07124733
	10-0813	NA	2000	Manitoba	Clinical	Blood	1/2a	7	7	LMACI.0118	LMAAI.0122			Complete	CP007171	PRJNA167879	SAMN07124734
Cheese C, 2002	10-4758	NA	2002	Quebec	Food	Cheese	1/2a	37	37	LMACI.0616	LMAAI.0818	17 cases: 11 hospitalized, 2 perinatal with premature births	Gaulin C, Currie A, Gravel G, Hamel M, Leblanc MA, Ramsay D, Bekal S: Summary of 11 years of enteric outbreak investigations and criteria to initiate an investigation, Province of Quebec, 2002 through 2012. J Food Prot 2014, 77(9):1563-1570.	Complete	CP007198	PRJNA167881	SAMN07124736
	10-4754	NA	2002	Quebec	Clinical	CSF	1/2a	37	37	LMACI.0616	LMAAI.0818			Complete	CP007197	PRJNA167880	SAMN07124735
Cheese A, 2002	02-1792	NA	2002	British Columbia	Food	Cheese	4b	1	1	LMACI.0023	LMAAI.0140	47 cases including: 2 meningitis and 2 perinatal bacteremia	[21]	Complete	CP007461	PRJNA167886	SAMN07124741
	02-1289	NA	2002	British Columbia	Clinical	Stool	4b	1	1	LMACI.0023	LMAAI.0140			Complete	CP007460	PRJNA167885	SAMN07124740
	02-1103	NA	2002	British Columbia	Clinical	CSF	4b	1	1	LMACI.0023	LMAAI.0140			Complete	CP007459	PRJNA167884	SAMN07124739
Cheese B, 2002	02-6680	NA	2002	British Columbia	Food	Cheese	4b	388	n/a	LMACI.0082	LMAAI.0017	86 febrile gastrointestinal cases	[21]	Complete	CP007462	PRJNA167888	SAMN07124742
	02-6679	NA	2002	British Columbia	Clinical	Stool	4b	388	n/a	LMACI.0082	LMAAI.0017			Complete	CP008821	PRJNA167887	SAMN02944848
Cheese D, 2008	10-0934	NA	2008	Quebec	Food	Cheese	1/2a	394	415	LMACI.0149	LMAAI.0265	39 cases including: 7 perinatal, 1 adult death, 1 stillborn	This study	Complete	CP007200	PRJNA167883	SAMN07124738
	10-0933	NA	2008	Quebec	Clinical	Blood	1/2a	394	415	LMACI.0149	LMAAI.0265			Complete	CP007199	PRJNA167882	SAMN07124737
Ready to eat (RTE) delimeat, 2008	08-6569	08-E01	2008	Ontario	Processing Environment	Food processing	1/2a	292	8	LMACI.0040	LMAAI.0001	57 cases including 24 listeriosis-confirmed deaths and 0 perinatal deaths	[8, 10] and this Study	Complete	CP006858	PRJNA167896	SAMN05771122
	08-7374	08-F02	2008	Ontario	Food	RTE Coorsh smoked meat	1/2a	292	8	LMACI.0001	LMAAI.0001			Complete	CP007010	PRJNA167898	SAMN07124749
	08-6056	08-F01	2008	Ontario	Food	RTE turkey meat	1/2a	292	8	LMACI.0040	LMAAI.0003			Complete	CP007009	PRJNA167895	SAMN07124748
	10-0814	08-F04	2008	Ontario	Food	RTE meat	1/2a	292	8	LMACI.0001	LMAAI.0001			Complete	CP008836	PRJNA167900	SAMN02873948
	10-0815	08-F05	2008	Saskatchewan	Food	RTE meat	1/2a	292	8	LMACI.0040	LMAAI.0001			Complete	CP006860	PRJNA167901	SAMN05771120
	08-5923[1]	08-C16	2008	Ontario	Clinical	Blood	1/2a	120	8	LMACI.0001	LMAAI.0001			Complete	CP001604	PRJNA36363	SAMN02603721
	08-7669[1]	08-C18	2008	Saskatchewan	Clinical	Blood	1/2a	120	8	LMACI.0001	LMAAI.0001			Complete	CP007011	PRJNA167899	SAMN07124750
	08-5578	08-C15	2008	Ontario	Clinical	Blood	1/2a	292	8	LMACI.0040	LMAAI.0001			Complete	CP001602/ CP001603	PRJNA36361	SAMN02603776
	08-6997	08-C17	2008	Ontario	Clinical	Blood	1/2a	292	8	LMACI.0040	LMAAI.0003			Complete	CP006859	PRJNA167897	SAMN05771121
	08-7362	08-E02	2008	Ontario	Processing Environment	Unknown	1/2a	120	8	LMACI.0001	LMAAI.0001			Draft	CP008765	PRJNA215186	SAMN02725201
	08-7363	08-E03	2008	Ontario	Processing Environment	Unknown	1/2a	120	8	LMACI.0001	LMAAI.0001			Draft	CP008766	PRJNA215187	SAMN02725202
	08-7364	08-E04	2008	Ontario	Processing Environment	Unknown	1/2a	120	8	LMACI.0001	LMAAI.0001			Draft	CP008767	PRJNA215188	SAMN02725203
Ready to eat (RTE) prosciutto ham, 2010	10-5024	10-F03	2010	Ontario	Clinical	RTE prosciutto	1/2a	120	8	LMACI.0001	LMAAI.0001	2 cases, 0 deaths	This study	Complete	CP008837	PRJNA167905	SAMN02725209
	10-1321	10-C24	2010	Ontario	Clinical	Blood	1/2a	120	8	LMACI.0001	LMAAI.0001			Complete	CP007018	PRJNA167904	SAMN07124752
	10-1046[2]	10-C22	2010	Ontario	Clinical	Blood	1/2a	120	8	LMACI.0001	LMAAI.0001			Complete	CP007017	PRJNA167902	SAMN07124751

	10-1047[2]	10-C23	2010	Ontario	Clinical	CSF	1/2a	120	8	LMACI.0001	LMAAI.0001			Complete	CP006861	PRJNA167903	SAMN05771119
Sporadic	88-0478	88-C01	1988	Ontario	Clinical	Blood	1/2a	120	8	LMACI.0001	LMAAI.0001	Unknown epidemiology	This study	Complete	CP006862	PRJNA167889	SAMN05771118
	88-1059	88-C02	1988	Newfoundland	Clinical	Blood	3a	120	8	LMACI.0001	LMAAI.0001			Draft	CP008770	PRJNA215191	SAMN02725206
	95-0093	95-C03	1995	Alberta	Clinical	Blood	1/2a	120	8	LMACI.0001	LMAAI.0001			Complete	CP007019	PRJNA167890	SAMN07124743
	98-0291	98-C04	1998	Ontario	Clinical	Blood	1/2a	120	8	LMACI.0001	LMAAI.0001			Draft	CP008771	PRJNA215192	SAMN02725207
	98-2035	98-C05	1998	Ontario	Clinical	Blood	1/2a	292	8	LMACI.0040	LMAAI.0003			Complete	CP007020	PRJNA167891	SAMN07124744
	99-6370	99-C06	1999	Newfoundland	Clinical	Blood	1/2a	292	8	LMACI.0040	LMAAI.0003			Complete	CP007021	PRJNA167892	SAMN07124745
	99-6871	99-C07	1999	Ontario	Clinical	Tissue	1/2a	120	8	LMACI.0001	LMAAI.0001			Draft	CP008772	PRJNA215193	SAMN02725208
	01-1280	01-C08	2001	Ontario	Clinical	Blood	1/2a	292	8	LMACI.0001	LMAAI.0001			Draft	CP006940	PRJNA215181	SAMN05771117
	01-1468	01-C09	2001	Newfoundland	Clinical	Brain tissue	1/2a	120	8	LMACI.0098	LMAAI.0081			Draft	CP007527	PRJNA215182	SAMN02693102
	01-5252	01-C10	2001	Manitoba	Clinical	Blood	1/2a	387	8	LMACI.0001	LMAAI.0003			Draft	CP007538	PRJNA215183	SAMN02725064
	01-6771	01-C11	2001	Ontario	Clinical	Vitreous	1/2a	120	8	LMACI.0001	LMAAI.0142			Draft	CP008703	PRJNA215184	SAMN02725199
	02-5993	02-C12	2002	Ontario	Clinical	Blood	1/2a	120	8	LMACI.0001	LMAAI.0001			Complete	CP007007	PRJNA167893	SAMN07124746
	03-5473	03-C13	2003	Ontario	Clinical	Blood	1/2a	120	8	LMACI.0001	LMAAI.0001			Draft	CP008773	PRJNA215185	SAMN02725200
	04-5457	04-C14	2004	Ontario	Clinical	Blood	1/2a	292	8	LMACI.0001	LMAAI.0001			Complete	CP007008	PRJNA167894	SAMN07124747
	10-0819	10-C21	2010	Ontario	Clinical	Blood	1/2a	8	8	LMACI.0618	LMAAI.0003			Draft	CP008768	PRJNA215189	SAMN02725204
	11-4254	11-C25	2011	British Columbia	Clinical	Blood	1/2a	120	8	LMACI.0573	LMAAI.0214			Draft	CP008769	PRJNA215190	SAMN02725205
Non-Outbreak Associated FPE[3]	10-5025	NA	2009	Ontario	Food	Fully cooked bacon flakes	1/2c	9	9	LMACI.0036	LMAAI.0658	Same establishment as 10-5025	This study	Complete	CP007194	PRJNA167906	SAMN07124753
	10-5026	NA	2009	Ontario	Processing environment	Composite swab: dicer exit and blade	1/2c	9	9	LMACI.0036	LMAAI.0658	Same establishment as 10-5026		Complete	CP007195	PRJNA167907	SAMN07124754
	10-5027	NA	2009	Ontario	Processing environment	Composite swab: chute, 2 trays, scoop, polybag liner, inside hopper, hopper leveling bar, belt top & bottom	3c	9	9	LMACI.0036	LMAAI.0863	Distinct establishment		Complete	CP007196	PRJNA167908	SAMN07124755

RTE, ready to eat; CSF, cerebrospinal fluid; FPE, Food Processing Equipment; PFGE, pulsed-field gele electrophoresis

Average depth of coverage from 115-1227x completed genomes (n=37) using Roche 454 and Illumina data, and from 46-171x (or >50x) for draft genomes (n=13) using GAIIx data. Genomes for 08-5578 and 08-5923 were published previously [10]

[1]Isolates 08-5923 and 08-7669 originally included in 2008 deli meat outbreak investigation deemed unlikely associated due to conflicting epidemiology and typing analyses [8]

[2]Same patient

[3]Common serotype in FPE but uncommon pathogen

Table S2A. Core hqSNV distance matrix for 39 *Listeria monocytogenes* isolates (2010 dataset)

Isolate	10-0933	10-0934	81-0592	10-0809	81-0558	81-0861	02-1103	02-1289	02-1792	10-5025	10-5026	10-5027	02-6680	02-6679	10-0812	10-0813	08-7669	08-5923
10-0933	0	0	109608	109609	109608	109609	109601	109601	109601	20278	20278	20278	109660	109659	20506	20505	20352	20345
10-0934	0	0	109608	109609	109608	109609	109601	109601	109601	20278	20278	20278	109660	109659	20506	20505	20352	20345
81-0592	109608	109608	0	13	14	18	142	142	142	109679	109679	109679	6665	6666	109190	109189	108762	108756
10-0809	109609	109609	13	0	15	21	145	145	145	109680	109680	109680	6668	6667	109191	109190	108763	108757
81-0558	109608	109608	14	15	0	20	144	144	144	109679	109679	109679	6667	6666	109190	109189	108760	108754
81-0861	109609	109609	18	21	20	0	148	148	148	109680	109680	109680	6669	6668	109191	109190	108761	108755
02-1289	109601	109601	142	145	144	148	0	0	0	109676	109676	109676	6664	6665	109186	109185	108759	108753
02-1103	109601	109601	142	145	144	148	0	0	0	109676	109676	109676	6664	6665	109186	109185	108759	108753
02-1792	109601	109601	142	145	144	148	0	0	0	109676	109676	109676	6664	6665	109186	109185	108759	108753
10-5025	20278	20278	109679	109680	109679	109680	109676	109676	109676	0	0	22	109772	109771	21142	21141	19049	19042
10-5026	20278	20278	109679	109680	109679	109680	109676	109676	109676	0	0	22	109772	109771	21142	21141	19049	19042
10-5027	20278	20278	109679	109680	109679	109680	109676	109676	109676	22	22	0	109772	109771	21144	21143	19051	19044
02-6679	109659	109659	6666	6667	6666	6668	6665	6665	6665	109771	109771	109771	1	0	109257	109256	108794	108788
02-6680	109660	109660	6665	6668	6667	6669	6664	6664	6664	109772	109772	109772	0	1	109258	109257	108795	108789
10-0812	20506	20506	109190	109191	109190	109191	109186	109186	109186	21142	21142	21144	109258	109257	0	1	21377	21370
10-0813	20505	20505	109189	109190	109189	109190	109185	109185	109185	21141	21141	21143	109257	109256	1	0	21376	21369
08-7669	20352	20352	108762	108763	108760	108761	108759	108759	108759	19049	19049	19051	108795	108794	21377	21376	0	27
08-5923	20345	20345	108756	108757	108754	108755	108753	108753	108753	19042	19042	19044	108789	108788	21370	21369	27	0
02-5993	20343	20343	108754	108755	108752	108753	108751	108751	108751	19040	19040	19042	108787	108786	21368	21367	25	18
08-6569	20342	20342	108754	108755	108752	108753	108751	108751	108751	19040	19040	19042	108787	108786	21367	21366	23	18
08-6997	20342	20342	108754	108755	108752	108753	108751	108751	108751	19040	19040	19042	108787	108786	21367	21366	23	18
08-7374	20342	20342	108754	108755	108752	108753	108751	108751	108751	19040	19040	19042	108787	108786	21367	21366	23	18
10-0814	20342	20342	108754	108755	108752	108753	108751	108751	108751	19040	19040	19042	108787	108786	21367	21366	23	18
10-1046	20341	20341	108753	108754	108751	108752	108750	108750	108750	19038	19038	19040	108786	108785	21366	21365	23	4
10-1047	20341	20341	108753	108754	108751	108752	108750	108750	108750	19038	19038	19040	108786	108785	21366	21365	23	4
10-1321	20341	20341	108753	108754	108751	108752	108750	108750	108750	19038	19038	19040	108786	108785	21366	21365	23	4
04-5457	20341	20341	108753	108754	108751	108752	108750	108750	108750	19039	19039	19041	108786	108785	21366	21365	22	17
08-5578	20341	20341	108753	108754	108751	108752	108750	108750	108750	19039	19039	19041	108786	108785	21366	21365	22	17
08-5578v3	20341	20341	108753	108754	108751	108752	108750	108750	108750	19039	19039	19041	108786	108785	21366	21365	22	17
08-6056	20341	20341	108753	108754	108751	108752	108750	108750	108750	19039	19039	19041	108786	108785	21366	21365	22	17
10-0815	20341	20341	108753	108754	108751	108752	108750	108750	108750	19039	19039	19041	108786	108785	21366	21365	22	17
10-5024	20343	20343	108753	108754	108751	108752	108750	108750	108750	19040	19040	19042	108786	108785	21368	21367	25	6
88-0478	20341	20341	108753	108754	108751	108752	108750	108750	108750	19038	19038	19040	108786	108785	21366	21365	23	16
98-2035	20341	20341	108753	108754	108751	108752	108750	108750	108750	19039	19039	19041	108786	108785	21366	21365	22	17
99-6370	20341	20341	108753	108754	108751	108752	108750	108750	108750	19039	19039	19041	108786	108785	21366	21365	22	17
95-0093	20339	20339	108751	108752	108749	108750	108748	108748	108748	19036	19036	19038	108784	108783	21362	21361	19	14
10-0810	109167	109167	9295	9296	9295	9298	9297	9297	9297	109290	109290	109290	8963	8962	108660	108659	108314	108308
10-0811	109170	109170	9294	9297	9296	9299	9296	9296	9296	109293	109293	109293	8962	8963	108663	108662	108317	108311
10-4758	22117	22117	108818	108819	108818	108819	108819	108819	108819	20524	20524	20526	108965	108964	22003	22002	20988	20981
10-4754	22116	22116	108817	108818	108817	108818	108818	108818	108818	20523	20523	20525	108964	108963	22002	22001	20987	20980

Isolate	02-5993	08-6569	08-6997	08-7374	10-0814	04-5457	08-5578	08-5578v3	08-6056	10-0815	10-1046	10-1047	10-1321	10-5024	88-0478	98-2035	99-6370	95-0093	10-0810	10-0811	10-4758	10-4754
10-0933	20343	20342	20342	20342	20342	20341	20341	20341	20341	20341	20341	20341	20341	20343	20341	20341	20341	20339	109167	109170	22117	22116
10-0934	20343	20342	20342	20342	20342	20341	20341	20341	20341	20341	20341	20341	20341	20343	20341	20341	20341	20339	109167	109170	22117	22116
81-0592	108754	108754	108754	108754	108754	108753	108753	108753	108753	108753	108753	108753	108753	108753	108753	108753	108753	108751	9295	9294	108818	108817
10-0809	108755	108755	108755	108755	108755	108754	108754	108754	108754	108754	108754	108754	108754	108754	108754	108754	108754	108752	9296	9297	108819	108818
81-0558	108752	108752	108752	108752	108752	108751	108751	108751	108751	108751	108751	108751	108751	108751	108751	108751	108751	108749	9295	9296	108818	108817
81-0861	108753	108753	108753	108753	108753	108752	108752	108752	108752	108752	108752	108752	108752	108752	108752	108752	108752	108750	9298	9299	108819	108818
02-1289	108751	108751	108751	108751	108751	108750	108750	108750	108750	108750	108750	108750	108750	108750	108750	108750	108750	108748	9297	9296	108819	108818
02-1103	108751	108751	108751	108751	108751	108750	108750	108750	108750	108750	108750	108750	108750	108750	108750	108750	108750	108748	9297	9296	108819	108818
02-1792	108751	108751	108751	108751	108751	108750	108750	108750	108750	108750	108750	108750	108750	108750	108750	108750	108750	108748	9297	9296	108819	108818
10-5025	19040	19040	19040	19040	19040	19039	19039	19039	19039	19039	19038	19038	19038	19040	19038	19039	19039	19036	109290	109293	20524	20523
10-5026	19040	19040	19040	19040	19040	19039	19039	19039	19039	19039	19038	19038	19038	19040	19038	19039	19039	19036	109290	109293	20524	20523
10-5027	19042	19042	19042	19042	19042	19041	19041	19041	19041	19041	19040	19040	19040	19042	19040	19041	19041	19038	109290	109293	20526	20525
02-6679	108786	108786	108786	108786	108786	108785	108785	108785	108785	108785	108785	108785	108785	108785	108785	108785	108785	108783	8962	8963	108964	108963
02-6680	108787	108787	108787	108787	108787	108786	108786	108786	108786	108786	108786	108786	108786	108786	108786	108786	108786	108784	8963	8962	108965	108964
10-0812	21368	21367	21367	21367	21367	21366	21366	21366	21366	21366	21366	21366	21366	21368	21366	21366	21366	21362	108660	108663	22003	22002
10-0813	21367	21366	21366	21366	21366	21365	21365	21365	21365	21365	21365	21365	21365	21367	21365	21365	21365	21361	108659	108662	22002	22001
08-7669	25	23	23	23	23	22	22	22	22	22	23	23	23	25	23	22	22	19	108314	108317	20988	20987
08-5923	18	18	18	18	18	17	17	17	17	17	4	4	4	6	16	17	17	14	108308	108311	20981	20980
02-5993	0	16	16	16	16	15	15	15	15	15	14	14	14	16	14	15	15	12	108306	108309	20979	20978
08-6569	16	0	2	4	2	3	1	1	1	1	14	14	14	16	14	3	3	10	108306	108309	20979	20978
08-6997	16	2	0	4	2	3	1	1	1	1	14	14	14	16	14	3	3	10	108306	108309	20979	20978
08-7374	16	4	4	0	4	3	3	3	3	3	14	14	14	16	14	3	3	10	108306	108309	20979	20978
10-0814	16	2	2	4	0	3	1	1	1	1	14	14	14	16	14	3	3	10	108306	108309	20979	20978
10-1046	14	14	14	14	14	13	13	13	13	13	0	0	0	2	12	13	13	10	108305	108308	20977	20976
10-1047	14	14	14	14	14	13	13	13	13	13	0	0	0	2	12	13	13	10	108305	108308	20977	20976
10-1321	14	14	14	14	14	13	13	13	13	13	0	0	0	2	12	13	13	10	108305	108308	20977	20976
04-5457	15	3	3	3	3	0	2	2	2	2	13	13	13	15	13	2	2	9	108305	108308	20978	20977
08-5578	15	1	1	3	1	2	0	0	0	0	13	13	13	15	13	2	2	9	108305	108308	20978	20977
08-5578v3	15	1	1	3	1	2	0	0	0	0	13	13	13	15	13	2	2	9	108305	108308	20978	20977
08-6056	15	1	1	3	1	2	0	0	0	0	13	13	13	15	13	2	2	9	108305	108308	20978	20977
10-0815	15	1	1	3	1	2	0	0	0	0	13	13	13	15	13	2	2	9	108305	108308	20978	20977
10-5024	16	16	16	16	16	15	15	15	15	15	2	2	2	0	14	15	15	12	108305	108308	20979	20978
88-0478	14	14	14	14	14	13	13	13	13	13	12	12	12	14	0	13	13	10	108305	108308	20977	20976
98-2035	15	3	3	3	3	2	2	2	2	2	13	13	13	15	13	0	2	9	108305	108308	20978	20977
99-6370	15	3	3	3	3	2	2	2	2	2	13	13	13	15	13	2	0	9	108305	108308	20978	20977
95-0093	12	10	10	10	10	9	9	9	9	9	10	10	10	12	10	9	9	0	108303	108306	20975	20974
10-0810	108306	108306	108306	108306	108306	108305	108305	108305	108305	108305	108305	108305	108305	108305	108305	108305	108305	108303	0	3	108402	108401
10-0811	108309	108309	108309	108309	108309	108308	108308	108308	108308	108308	108308	108308	108308	108308	108308	108308	108308	108306	3	0	108405	108404
10-4758	20979	20979	20979	20979	20979	20978	20978	20978	20978	20978	20977	20977	20977	20979	20977	20978	20978	20975	108402	108405	0	1
10-4754	20978	20978	20978	20978	20978	20977	20977	20977	20977	20977	20976	20976	20976	20978	20976	20977	20977	20974	108401	108404	1	0

Table S2B. Core hqSNV distance matrix for 39 *Listeria monocytogenes* isolates (2014 dataset)

Isolate	10-0933	10-0934	81-0592	10-0809	81-0558	81-0861	02-1289	02-1103	02-1792	10-5025	10-5026	10-5027	02-6679	02-6680	10-0812	10-0813	08-7669	08-5923	02-5993	08-6569
10-0933	0	0	115441	115440	115440	115438	115431	115430	115430	21096	21096	21095	115397	115396	21294	21293	21594	21588	21586	21585
10-0934	0	0	115441	115440	115440	115438	115431	115430	115430	21096	21096	21095	115397	115396	21294	21293	21594	21588	21586	21585
81-0592	115441	115441	0	15	13	17	147	148	148	115400	115400	115399	6862	6863	114888	114887	114466	114461	114459	114459
10-0809	115440	115440	15	0	12	18	148	149	149	115399	115399	115398	6861	6860	114885	114884	114465	114460	114458	114458
81-0558	115440	115440	13	12	0	14	146	147	147	115399	115399	115398	6859	6860	114885	114884	114465	114460	114458	114458
81-0861	115438	115438	17	18	14	0	150	149	149	115397	115397	115396	6863	6864	114883	114882	114463	114458	114456	114456
02-1289	115431	115431	147	148	146	150	0	1	3	115394	115394	115393	6858	6859	114879	114878	114462	114457	114455	114455
02-1103	115430	115430	148	149	147	149	1	0	2	115393	115393	115392	6859	6860	114878	114877	114461	114456	114454	114454
02-1792	115430	115430	148	149	147	149	3	2	0	115393	115393	115392	6859	6860	114878	114877	114461	114456	114454	114454
10-5025	21096	21096	115400	115399	115399	115397	115394	115393	115393	0	0	23	115386	115385	21845	21844	20046	20040	20038	20038
10-5026	21096	21096	115400	115399	115399	115397	115394	115393	115393	0	0	23	115386	115385	21845	21844	20046	20040	20038	20038
10-5027	21095	21095	115399	115398	115398	115396	115393	115392	115392	23	23	0	115385	115384	21846	21845	20047	20041	20039	20039
02-6679	115397	115397	6862	6861	6859	6863	6858	6859	6859	115386	115386	115385	0	1	114845	114844	114395	114390	114388	114388
02-6680	115396	115396	6863	6860	6860	6864	6859	6860	6860	115385	115385	115384	1	0	114844	114843	114394	114389	114387	114387
10-0812	21294	21294	114888	114885	114885	114883	114879	114878	114878	21845	21845	21846	114845	114844	0	1	22450	22444	22442	22441
10-0813	21293	21293	114887	114884	114884	114882	114878	114877	114877	21844	21844	21845	114844	114843	1	0	22449	22443	22441	22440
08-7669	21594	21594	114466	114465	114465	114463	114462	114461	114461	20046	20046	20047	114395	114394	22450	22449	0	28	24	22
08-5923	21588	21588	114461	114460	114460	114458	114457	114456	114456	20040	20040	20041	114390	114389	22444	22443	28	0	20	20
02-5993	21586	21586	114459	114458	114458	114456	114455	114454	114454	20038	20038	20039	114388	114387	22442	22441	24	20	0	16
08-6569	21585	21585	114459	114458	114458	114456	114455	114454	114454	20038	20038	20039	114388	114387	22441	22440	22	20	16	0
08-6997	21585	21585	114459	114458	114458	114456	114455	114454	114454	20038	20038	20039	114388	114387	22441	22440	22	20	16	2
08-7374	21585	21585	114459	114458	114458	114456	114455	114454	114454	20038	20038	20039	114388	114387	22441	22440	22	20	16	4
10-0814	21585	21585	114459	114458	114458	114456	114455	114454	114454	20038	20038	20039	114388	114387	22441	22440	22	20	16	2
10-1046	21585	21585	114459	114458	114458	114456	114455	114454	114454	20037	20037	20038	114388	114387	22441	22440	25	5	17	17
10-1047	21585	21585	114459	114458	114458	114456	114455	114454	114454	20037	20037	20038	114388	114387	22441	22440	25	5	17	17
10-1321	21585	21585	114459	114458	114458	114456	114455	114454	114454	20037	20037	20038	114388	114387	22441	22440	25	5	17	17
04-5457	21584	21584	114458	114457	114457	114455	114454	114453	114453	20037	20037	20038	114387	114386	22440	22439	21	19	15	3
08-5578	21584	21584	114458	114457	114457	114455	114454	114453	114453	20037	20037	20038	114387	114386	22440	22439	21	19	15	1
08-5578v3	21584	21584	114458	114457	114457	114455	114454	114453	114453	20037	20037	20038	114387	114386	22440	22439	21	19	15	1
08-6056	21584	21584	114458	114457	114457	114455	114454	114453	114453	20037	20037	20038	114387	114386	22440	22439	21	19	15	1
10-0815	21584	21584	114458	114457	114457	114455	114454	114453	114453	20037	20037	20038	114387	114386	22440	22439	21	19	15	1
10-5024	21586	21586	114458	114457	114457	114455	114454	114453	114453	20038	20038	20039	114387	114386	22442	22441	26	6	18	18
88-0478	21584	21584	114458	114457	114457	114455	114454	114453	114453	20036	20036	20037	114387	114386	22440	22439	22	18	14	14
98-2035	21584	21584	114458	114457	114457	114455	114454	114453	114453	20037	20037	20038	114387	114386	22440	22439	21	19	15	3
99-6370	21584	21584	114458	114457	114457	114455	114454	114453	114453	20037	20037	20038	114387	114386	22440	22439	21	19	15	3
95-0093	21582	21582	114456	114455	114455	114453	114452	114451	114451	20034	20034	20035	114385	114384	22436	22435	18	16	12	10
10-0810	114343	114343	10103	10100	10100	10104	10100	10101	10101	114414	114414	114413	9769	9768	113760	113759	113416	113411	113409	113409
10-0811	114342	114342	10104	10103	10101	10101	10101	10102	10102	114413	114413	114412	9770	9771	113759	113758	113415	113410	113408	113408
10-4758	23243	23243	114343	114342	114342	114340	114339	114338	114338	21490	21490	21491	114372	114371	22959	22958	22107	22101	22099	22099
10-4754	23242	23242	114342	114341	114341	114339	114338	114337	114337	21489	21489	21490	114371	114370	22958	22957	22106	22100	22098	22098

Isolate	08-6997	08-7374	10-0814	10-1046	10-1047	10-1321	04-5457	08-5578	08-5578v3	08-6056	10-0815	10-5024	88-0478	98-2035	99-6370	95-0093	10-0810	10-0811	10-4758	10-4754
10-0933	21585	21585	21585	21585	21585	21585	21584	21584	21584	21584	21584	21586	21584	21584	21584	21582	114343	114342	23243	23242
10-0934	21585	21585	21585	21585	21585	21585	21584	21584	21584	21584	21584	21586	21584	21584	21584	21582	114343	114342	23243	23242
81-0592	114459	114459	114459	114459	114459	114459	114458	114458	114458	114458	114458	114458	114458	114458	114458	114456	10103	10104	114343	114342
10-0809	114458	114458	114458	114458	114458	114458	114457	114457	114457	114457	114457	114457	114457	114457	114457	114455	10100	10103	114342	114341
81-0558	114458	114458	114458	114458	114458	114458	114457	114457	114457	114457	114457	114457	114457	114457	114457	114455	10100	10101	114342	114341
81-0861	114456	114456	114456	114456	114456	114456	114455	114455	114455	114455	114455	114455	114455	114455	114455	114453	10104	10101	114340	114339
02-1289	114455	114455	114455	114455	114455	114455	114454	114454	114454	114454	114454	114454	114454	114454	114454	114452	10100	10101	114339	114338
02-1103	114454	114454	114454	114454	114454	114454	114453	114453	114453	114453	114453	114453	114453	114453	114453	114451	10101	10102	114338	114337
02-1792	114454	114454	114454	114454	114454	114454	114453	114453	114453	114453	114453	114453	114453	114453	114453	114451	10101	10102	114338	114337
10-5025	20038	20038	20038	20037	20037	20037	20037	20037	20037	20037	20037	20038	20036	20037	20037	20034	114414	114413	21490	21489
10-5026	20038	20038	20038	20037	20037	20037	20037	20037	20037	20037	20037	20038	20036	20037	20037	20034	114414	114413	21490	21489
10-5027	20039	20039	20039	20038	20038	20038	20038	20038	20038	20038	20038	20039	20037	20038	20038	20035	114413	114412	21491	21490
02-6679	114388	114388	114388	114388	114388	114388	114387	114387	114387	114387	114387	114387	114387	114387	114387	114385	9769	9770	114372	114371
02-6680	114387	114387	114387	114387	114387	114387	114386	114386	114386	114386	114386	114386	114386	114386	114386	114384	9768	9771	114371	114370
10-0812	22441	22441	22441	22441	22441	22441	22440	22440	22440	22440	22440	22442	22440	22440	22440	22436	113760	113759	22959	22958
10-0813	22440	22440	22440	22440	22440	22440	22439	22439	22439	22439	22439	22441	22439	22439	22439	22435	113759	113758	22958	22957
08-7669	22	22	22	25	25	25	21	21	21	21	21	26	22	21	21	18	113416	113415	22107	22106
08-5923	20	20	20	5	5	5	19	19	19	19	19	6	18	19	19	16	113411	113410	22101	22100
02-5993	16	16	16	17	17	17	15	15	15	15	15	18	14	15	15	12	113409	113408	22099	22098
08-6569	2	4	2	17	17	17	3	1	1	1	1	18	14	3	3	10	113409	113408	22099	22098
08-6997	0	4	2	17	17	17	3	1	1	1	1	18	14	3	3	10	113409	113408	22099	22098
08-7374	4	0	4	17	17	17	3	3	3	3	3	18	14	3	3	10	113409	113408	22099	22098
10-0814	2	4	0	17	17	17	3	1	1	1	1	18	14	3	3	10	113409	113408	22099	22098
10-1046	17	17	17	0	0	0	16	16	16	16	16	3	15	16	16	13	113409	113408	22098	22097
10-1047	17	17	17	0	0	0	16	16	16	16	16	3	15	16	16	13	113409	113408	22098	22097
10-1321	17	17	17	0	0	0	16	16	16	16	16	3	15	16	16	13	113409	113408	22098	22097
04-5457	3	3	3	16	16	16	0	2	2	2	2	17	13	2	2	9	113408	113407	22098	22097
08-5578	1	3	1	16	16	16	2	0	0	0	0	17	13	2	2	9	113408	113407	22098	22097
08-5578v3	1	3	1	16	16	16	2	0	0	0	0	17	13	2	2	9	113408	113407	22098	22097
08-6056	1	3	1	16	16	16	2	0	0	0	0	17	13	2	2	9	113408	113407	22098	22097
10-0815	1	3	1	16	16	16	2	0	0	0	0	17	13	2	2	9	113408	113407	22098	22097
10-5024	18	18	18	3	3	3	17	17	17	17	17	0	16	17	17	14	113408	113407	22099	22098
88-0478	14	14	14	15	15	15	13	13	13	13	13	16	0	13	13	10	113408	113407	22097	22096
98-2035	3	3	3	16	16	16	2	2	2	2	2	17	13	0	2	9	113408	113407	22098	22097
99-6370	3	3	3	16	16	16	2	2	2	2	2	17	13	2	0	9	113408	113407	22098	22097
95-0093	10	10	10	13	13	13	9	9	9	9	9	14	10	9	9	0	113406	113405	22095	22094
10-0810	113409	113409	113409	113409	113409	113409	113408	113408	113408	113408	113408	113408	113408	113408	113408	113406	0	5	113332	113331
10-0811	113408	113408	113408	113408	113408	113408	113407	113407	113407	113407	113407	113407	113407	113407	113407	113405	5	0	113331	113330
10-4758	22099	22099	22099	22098	22098	22098	22098	22098	22098	22098	22098	22099	22097	22098	22098	22095	113332	113331	0	1
10-4754	22098	22098	22098	22097	22097	22097	22097	22097	22097	22097	22097	22098	22096	22097	22097	22094	113331	113330	1	0

Table S3. Species-wide core hqSNV distance matrix.

Clonal Complex	Outbreak Investigation	Strain (2008 deli meat investigation)	Isolate	10-0810	10-0811	02-6679	02-6680	02-1289	02-1103	02-1792	10-0809	81-0592	81-0558	81-0861	10-0933	10-0934	10-0812	10-0813	10-4758	10-4754	10-5027	10-5025	10-5026	10-1046	10-1047	10-1321	10-5024	08-6569	08-5578	08-6056	10-0815	08-6997	10-0814	08-7374	08-7362	08-7363	08-7364	08-7669	08-5923	04-5457	98-2035	99-6370	98-0291	01-1280	95-0093	88-0478	01-5252	02-5993	01-1468	03-5473	01-6771	99-6871	88-1059	11-4254	10-0819	
				C	F	C	F	C	C	F	C	C	C	F	C	F	F	C	F	C	FPE	FPE	FPE	C	C	C	F	FPE	C	F	F	C	F	F	FPE	FPE	FPE	C	C	C	C	C	C	C	C	C	C	C	C	C	C	C	C	C	C	#Isolate Type
5			10-0810	0	5	9759	9758	####	####	####	####	####	####	####	####	####	####	####	####	####	####	####	####	####	####	####	####	####	####	####	####	####	####	####	####	####	####	####	####	####	####	####	####	####	####	####	####	####	####	####	####	####	####	####	####	
			10-0811	5	0	9760	9761	####	####	####	####	####	####	####	####	####	####	####	####	####	####	####	####	####	####	####	####	####	####	####	####	####	####	####	####	####	####	####	####	####	####	####	####	####	####	####	####	####	####	####	####	####	####	####	####	
n/a	2002 Cheese B		02-6679	9759	9760	0	1	6853	6854	6854	6856	6857	6854	6858	####	####	####	####	####	####	####	####	####	####	####	####	####	####	####	####	####	####	####	####	####	####	####	####	####	####	####	####	####	####	####	####	####	####	####	####	####	####	####	####	####	
			02-6680	9758	9761	1	0	6854	6855	6855	6855	6858	6855	6859	####	####	####	####	####	####	####	####	####	####	####	####	####	####	####	####	####	####	####	####	####	####	####	####	####	####	####	####	####	####	####	####	####	####	####	####	####	####	####	####	####	
			02-1289	####	####	6853	6854	0	1	3	148	147	146	150	####	####	####	####	####	####	####	####	####	####	####	####	####	####	####	####	####	####	####	####	####	####	####	####	####	####	####	####	####	####	####	####	####	####	####	####	####	####	####	####	####	
	2002 Cheese A		02-1103	####	####	6854	6855	1	0	2	149	148	147	149	####	####	####	####	####	####	####	####	####	####	####	####	####	####	####	####	####	####	####	####	####	####	####	####	####	####	####	####	####	####	####	####	####	####	####	####	####	####	####	####	####	
			02-1792	####	####	6854	6855	3	2	0	149	148	147	149	####	####	####	####	####	####	####	####	####	####	####	####	####	####	####	####	####	####	####	####	####	####	####	####	####	####	####	####	####	####	####	####	####	####	####	####	####	####	####	####	####	
1			10-0809	####	####	6856	6855	148	149	149	0	15	12	18	####	####	####	####	####	####	####	####	####	####	####	####	####	####	####	####	####	####	####	####	####	####	####	####	####	####	####	####	####	####	####	####	####	####	####	####	####	####	####	####	####	
	1981 Coleslaw		81-0592	####	####	6857	6858	147	148	148	15	0	13	17	####	####	####	####	####	####	####	####	####	####	####	####	####	####	####	####	####	####	####	####	####	####	####	####	####	####	####	####	####	####	####	####	####	####	####	####	####	####	####	####	####	
			81-0558	####	####	6854	6855	146	147	147	12	13	0	14	####	####	####	####	####	####	####	####	####	####	####	####	####	####	####	####	####	####	####	####	####	####	####	####	####	####	####	####	####	####	####	####	####	####	####	####	####	####	####	####	####	
			81-0861	####	####	6858	6859	150	149	149	18	17	14	0	####	####	####	####	####	####	####	####	####	####	####	####	####	####	####	####	####	####	####	####	####	####	####	####	####	####	####	####	####	####	####	####	####	####	####	####	####	####	####	####	####	
415	2008 Cheese D		10-0933	####	####	####	####	####	####	####	####	####	####	####	0	0	####	####	####	####	####	####	####	####	####	####	####	####	####	####	####	####	####	####	####	####	####	####	####	####	####	####	####	####	####	####	####	####	####	####	####	####	####	####	####	
			10-0934	####	####	####	####	####	####	####	####	####	####	####	0	0	####	####	####	####	####	####	####	####	####	####	####	####	####	####	####	####	####	####	####	####	####	####	####	####	####	####	####	####	####	####	####	####	####	####	####	####	####	####	####	
7	2000 Whipping Cream		10-0812	####	####	####	####	####	####	####	####	####	####	####	####	####	0	1	####	####	####	####	####	####	####	####	####	####	####	####	####	####	####	####	####	####	####	####	####	####	####	####	####	####	####	####	####	####	####	####	####	####	####	####	####	
			10-0813	####	####	####	####	####	####	####	####	####	####	####	####	####	1	0	####	####	####	####	####	####	####	####	####	####	####	####	####	####	####	####	####	####	####	####	####	####	####	####	####	####	####	####	####	####	####	####	####	####	####	####	####	
37	2008 Cheese C		10-4758	####	####	####	####	####	####	####	####	####	####	####	####	####	####	####	0	1	####	####	####	####	####	####	####	####	####	####	####	####	####	####	####	####	####	####	####	####	####	####	####	####	####	####	####	####	####	####	####	####	####	####	####	
			10-4754	####	####	####	####	####	####	####	####	####	####	####	####	####	####	####	1	0	####	####	####	####	####	####	####	####	####	####	####	####	####	####	####	####	####	####	####	####	####	####	####	####	####	####	####	####	####	####	####	####	####	####	####	
			10-5027	####	####	####	####	####	####	####	####	####	####	####	####	####	####	####	####	####	0	23	23	####	####	####	####	####	####	####	####	####	####	####	####	####	####	####	####	####	####	####	####	####	####	####	####	####	####	####	####	####	####	####	####	
9			10-5025	####	####	####	####	####	####	####	####	####	####	####	####	####	####	####	####	####	23	0	0	####	####	####	####	####	####	####	####	####	####	####	####	####	####	####	####	####	####	####	####	####	####	####	####	####	####	####	####	####	####	####	####	
			10-5026	####	####	####	####	####	####	####	####	####	####	####	####	####	####	####	####	####	23	0	0	####	####	####	####	####	####	####	####	####	####	####	####	####	####	####	####	####	####	####	####	####	####	####	####	####	####	####	####	####	####	####	####	
			10-1046	####	####	####	####	####	####	####	####	####	####	####	####	####	####	####	####	####	####	####	####	0	0	0	3	17	16	16	16	17	17	17	18	18	18	25	5	16	16	16	18	17	13	15	17	17	24	21	24	24	22	82	124	
	2010 Prosciutto		10-1047	####	####	####	####	####	####	####	####	####	####	####	####	####	####	####	####	####	####	####	####	0	0	0	3	17	16	16	16	17	17	17	18	18	18	25	5	16	16	16	18	17	13	15	17	17	24	21	24	24	22	82	124	
			10-1321	####	####	####	####	####	####	####	####	####	####	####	####	####	####	####	####	####	####	####	####	0	0	0	3	17	16	16	16	17	17	17	18	18	18	25	5	16	16	16	18	17	13	15	17	17	24	21	24	24	22	82	124	
			10-5024	####	####	####	####	####	####	####	####	####	####	####	####	####	####	####	####	####	####	####	####	3	3	3	0	18	17	17	17	18	18	18	19	19	19	26	6	17	17	17	19	18	14	16	18	18	25	22	25	25	23	83	125	
			08-6569	####	####	####	####	####	####	####	####	####	####	####	####	####	####	####	####	####	####	####	####	17	17	17	18	0	1	1	1	2	2	4	15	15	15	22	20	3	3	3	5	4	10	14	16	16	21	18	25	25	21	83	125	
			08-5578	####	####	####	####	####	####	####	####	####	####	####	####	####	####	####	####	####	####	####	####	16	16	16	17	1	0	0	0	1	1	3	14	14	14	21	19	2	2	2	4	3	9	13	15	15	20	17	24	24	20	82	124	
		1	08-6056	####	####	####	####	####	####	####	####	####	####	####	####	####	####	####	####	####	####	####	####	16	16	16	17	1	0	0	0	1	1	3	14	14	14	21	19	2	2	2	4	3	9	13	15	15	20	17	24	24	20	82	124	
			10-0815	####	####	####	####	####	####	####	####	####	####	####	####	####	####	####	####	####	####	####	####	16	16	16	17	1	0	0	0	1	1	3	14	14	14	21	19	2	2	2	4	3	9	13	15	15	20	17	24	24	20	82	124	
			08-6997	####	####	####	####	####	####	####	####	####	####	####	####	####	####	####	####	####	####	####	####	17	17	17	18	2	1	1	1	0	2	4	15	15	15	22	20	3	3	3	5	4	10	14	16	16	21	18	25	25	21	83	125	
	2008 deli meat	2	10-0814	####	####	####	####	####	####	####	####	####	####	####	####	####	####	####	####	####	####	####	####	17	17	17	18	2	1	1	1	2	0	4	15	15	15	22	20	3	3	3	5	4	10	14	16	16	21	18	25	25	21	83	125	
		3	08-7374	####	####	####	####	####	####	####	####	####	####	####	####	####	####	####	####	####	####	####	####	17	17	17	18	4	3	3	3	4	4	0	15	15	15	22	20	3	3	3	5	4	10	14	16	16	21	18	25	25	21	83	125	
			08-7362	####	####	####	####	####	####	####	####	####	####	####	####	####	####	####	####	####	####	####	####	18	18	18	19	15	14	14	14	15	15	15	0	0	0	23	21	14	14	14	16	15	11	15	17	17	22	19	26	26	22	84	126	
		4	08-7363	####	####	####	####	####	####	####	####	####	####	####	####	####	####	####	####	####	####	####	####	18	18	18	19	15	14	14	14	15	15	15	0	0	0	23	21	14	14	14	16	15	11	15	17	17	22	19	26	26	22	84	126	
			08-7364	####	####	####	####	####	####	####	####	####	####	####	####	####	####	####	####	####	####	####	####	18	18	18	19	15	14	14	14	15	15	15	0	0	0	23	21	14	14	14	16	15	11	15	17	17	22	19	26	26	22	84	126	
		5	08-7669*	####	####	####	####	####	####	####	####	####	####	####	####	####	####	####	####	####	####	####	####	25	25	25	26	22	21	21	21	22	22	22	23	23	23	0	28	21	21	21	23	22	18	22	24	24	29	26	33	33	29	91	133	
8		6	08-5923*	####	####	####	####	####	####	####	####	####	####	####	####	####	####	####	####	####	####	####	####	5	5	5	6	20	19	19	19	20	20	20	21	21	21	28	0	19	19	19	21	20	16	18	20	20	27	24	27	27	25	85	127	
			04-5457	####	####	####	####	####	####	####	####	####	####	####	####	####	####	####	####	####	####	####	####	16	16	16	17	3	2	2	2	3	3	3	14	14	14	21	19	0	2	2	4	3	9	13	15	15	20	17	24	24	20	82	124	
			98-2035	####	####	####	####	####	####	####	####	####	####	####	####	####	####	####	####	####	####	####	####	16	16	16	17	3	2	2	2	3	3	3	14	14	14	21	19	2	0	2	4	3	9	13	15	15	20	17	24	24	20	82	124	
			99-6370	####	####	####	####	####	####	####	####	####	####	####	####	####	####	####	####	####	####	####	####	16	16	16	17	3	2	2	2	3	3	3	14	14	14	21	19	2	2	0	4	3	9	13	15	15	20	17	24	24	20	82	124	
			98-0291	####	####	####	####	####	####	####	####	####	####	####	####	####	####	####	####	####	####	####	####	18	18	18	19	5	4	4	4	5	5	5	16	16	16	23	21	4	4	4	0	5	11	15	17	17	22	19	26	26	22	84	126	
			01-1280	####	####	####	####	####	####	####	####	####	####	####	####	####	####	####	####	####	####	####	####	17	17	17	18	4	3	3	3	4	4	4	15	15	15	22	20	3	3	3	5	0	10	14	16	16	21	18	25	25	21	83	125	
			95-0093	####	####	####	####	####	####	####	####	####	####	####	####	####	####	####	####	####	####	####	####	13	13	13	14	10	9	9	9	10	10	10	11	11	11	18	16	9	9	9	11	10	0	10	12	12	13	10	21	21	17	79	121	
			88-0478	####	####	####	####	####	####	####	####	####	####	####	####	####	####	####	####	####	####	####	####	15	15	15	16	14	13	13	13	14	14	14	15	15	15	22	18	13	13	13	15	14	10	0	14	14	21	18	23	23	19	81	123	
	Sporadic		01-5252	####	####	####	####	####	####	####	####	####	####	####	####	####	####	####	####	####	####	####	####	17	17	17	18	16	15	15	15	16	16	16	17	17	17	24	20	15	15	15	17	16	12	14	0	4	23	20	25	25	17	83	125	
			02-5993	####	####	####	####	####	####	####	####	####	####	####	####	####	####	####	####	####	####	####	####	17	17	17	18	16	15	15	15	16	16	16	17	17	17	24	20	15	15	15	17	16	12	14	4	0	23	20	25	25	17	83	125	
			01-1468	####	####	####	####	####	####	####	####	####	####	####	####	####	####	####	####	####	####	####	####	24	24	24	25	21	20	20	20	21	21	21	22	22	22	29	27	20	20	20	22	21	13	21	23	23	0	15	32	32	28	90	132	
			03-5473	####	####	####	####	####	####	####	####	####	####	####	####	####	####	####	####	####	####	####	####	21	21	21	22	18	17	17	17	18	18	18	19	19	19	26	24	17	17	17	19	18	10	18	20	20	15	0	29	29	25	87	129	
			01-6771	####	####	####	####	####	####	####	####	####	####	####	####	####	####	####	####	####	####	####	####	24	24	24	25	25	24	24	24	25	25	25	26	26	26	33	27	24	24	24	26	25	21	23	25	25	32	29	0	6	30	90	132	
			99-6871	####	####	####	####	####	####	####	####	####	####	####	####	####	####	####	####	####	####	####	####	24	24	24	25	25	24	24	24	25	25	25	26	26	26	33	27	24	24	24	26	25	21	23	25	25	32	29	6	0	30	90	132	
			88-1059	####	####	####	####	####	####	####	####	####	####	####	####	####	####	####	####	####	####	####	####	22	22	22	23	21	20	20	20	21	21	21	22	22	22	29	25	20	20	20	22	21	17	19	17	17	28	25	30	30	0	88	130	
			11-4254	####	####	####	####	####	####	####	####	####	####	####	####	####	####	####	####	####	####	####	####	82	82	82	83	83	82	82	82	83	83	83	84	84	84	91	85	82	82	82	84	83	79	81	83	83	90	87	90	90	88	0	150	
			10-0819	####	####	####	####	####	####	####	####	####	####	####	####	####	####	####	####	####	####	####	####	124	124	124	125	125	124	124	124	125	125	125	126	126	126	133	127	124	124	124	126	125	121	123	125	125	132	129	132	132	130	150	0	

[#]Isolate types: C, Clinical; F, Food; FPE, Food processing environment.

*Isolates origincally included in outbreak investigation due to PFGE patterns that were later excluded due to contrary epidemiological evidence.

Note: Outbreak isolates without epidemiological links to outbreak were omitted from pairwise analysis (Figure 2). Epidemiologically-related pairs (green); pairs with known no epidemiolgical link belonging to the same CC (blue); pairs with known no epidemiolgical link belonging to a different CC (red);

Table S4. Lineage I core hqSNV distance matrix

Clonal Complex	Outbreak Investigation	Isolate	10-0810	10-0811	02-6679	02-6680	02-1103	02-1289	02-1792	81-0592	10-0809	81-0861	81-0558
		#Isolate Type	C	F	F	C	C	C	F	C	C	F	C
5		**10-0810**	0	1	11307	11307	11443	11443	11443	11450	11449	11449	11446
5		**10-0811**	1	0	11308	11308	11444	11444	11444	11451	11450	11450	11447
n/a	2002 Cheese B	**02-6679**	11307	11308	0	0	7928	7928	7928	7934	7933	7933	7930
n/a		**02-6680**	11307	11308	0	0	7928	7928	7928	7934	7933	7933	7930
		**02-1103**	11443	11444	7928	7928	0	0	0	165	164	164	161
	2002 Cheese A	**02-1289**	11443	11444	7928	7928	0	0	0	165	164	164	161
		**02-1792**	11443	11444	7928	7928	0	0	0	165	164	164	161
1		**81-0592**	11450	11451	7934	7934	165	165	165	0	17	17	14
	1981 Coleslaw	**10-0809**	11449	11450	7933	7933	164	164	164	17	0	16	13
		**81-0861**	11449	11450	7933	7933	164	164	164	17	16	0	11
		**81-0558**	11446	11447	7930	7930	161	161	161	14	13	11	0

#Isolate types: C, Clinical; F, Food; FPE, Food processing environment.

Note: Outbreak isolates without epidemiological links to outbreak were omitted from pairwise analysis (Figure 2). Epidemiologically-realted pairs (green); pairs with known no epidemiolgical link belonging to the same CC (blue); pairs with known no epidemiolgical link belonging to a different CC (red);

Table S5. Lineage II core hqSNV distance matrix

Clonal Complex	Outbreak Investigation	Strain (2008 deli meat investigation)	Isolate	10-5027	10-5025	10-5026	10-0933	10-0934	10-4758	10-4754	10-0812	10-0813	10-1321	10-1046	10-1047	10-5024	08-7362	08-7363	08-7364	08-6569	08-6997	08-5578	08-6056	10-0815	10-0814	08-7374	08-7669	08-5923	98-0291	01-1280	04-5457	99-6370	98-2035	95-0093	88-0478	01-5252	02-5993	88-1059	03-5473	10-0819	11-4254	01-1468	01-6771	99-6871
			#Isolate Type	FPE	FPE	FPE	C	F	F	C	F	C	C	C	C	F	FPE	FPE	FPE	FPE	C	C	C	C	C	C	C	C	C	C	C	C	C	C	C	C	C	C	C	C	C	C	C	C
			10-5027	0	27	27	####	####	####	####	####	####	####	####	####	####	####	####	####	####	####	####	####	####	####	####	####	####	####	####	####	####	####	####	####	####	####	####	####	####	####	####	####	####
9			10-5025	27	0	0	####	####	####	####	####	####	####	####	####	####	####	####	####	####	####	####	####	####	####	####	####	####	####	####	####	####	####	####	####	####	####	####	####	####	####	####	####	####
			10-5026	27	0	0	####	####	####	####	####	####	####	####	####	####	####	####	####	####	####	####	####	####	####	####	####	####	####	####	####	####	####	####	####	####	####	####	####	####	####	####	####	####
415	2008 Cheese D		10-0933	####	####	####	0	0	####	####	####	####	####	####	####	####	####	####	####	####	####	####	####	####	####	####	####	####	####	####	####	####	####	####	####	####	####	####	####	####	####	####	####	####
			10-0934	####	####	####	0	0	####	####	####	####	####	####	####	####	####	####	####	####	####	####	####	####	####	####	####	####	####	####	####	####	####	####	####	####	####	####	####	####	####	####	####	####
37	2008 Cheese C		10-4758	####	####	####	####	####	0	1	####	####	####	####	####	####	####	####	####	####	####	####	####	####	####	####	####	####	####	####	####	####	####	####	####	####	####	####	####	####	####	####	####	####
			10-4754	####	####	####	####	####	1	0	####	####	####	####	####	####	####	####	####	####	####	####	####	####	####	####	####	####	####	####	####	####	####	####	####	####	####	####	####	####	####	####	####	####
7	2000 Whipping Cream		10-0812	####	####	####	####	####	####	####	0	3	####	####	####	####	####	####	####	####	####	####	####	####	####	####	####	####	####	####	####	####	####	####	####	####	####	####	####	####	####	####	####	####
			10-0813	####	####	####	####	####	####	####	3	0	####	####	####	####	####	####	####	####	####	####	####	####	####	####	####	####	####	####	####	####	####	####	####	####	####	####	####	####	####	####	####	####
			10-1321	####	####	####	####	####	####	####	####	####	0	1	1	5	23	23	23	22	22	21	21	21	22	22	31	7	23	22	21	21	19	16	18	22	22	25	24	138	97	29	28	28
	2010 Prosciutto		10-1046	####	####	####	####	####	####	####	####	####	1	0	0	4	22	22	22	21	21	20	20	20	21	21	30	6	22	21	20	20	18	15	17	21	21	24	23	137	96	28	27	27
			10-1047	####	####	####	####	####	####	####	####	####	1	0	0	4	22	22	22	21	21	20	20	20	21	21	30	6	22	21	20	20	18	15	17	21	21	24	23	137	96	28	27	27
			10-5024	####	####	####	####	####	####	####	####	####	5	4	4	0	22	22	22	21	21	20	20	20	21	21	30	6	22	21	20	20	18	15	17	21	21	24	23	137	96	28	27	27
			08-7362	####	####	####	####	####	####	####	####	####	23	22	22	22	0	0	0	19	19	18	18	18	19	19	28	24	20	19	18	18	16	13	17	21	21	24	21	139	98	26	29	29
		4	08-7363	####	####	####	####	####	####	####	####	####	23	22	22	22	0	0	0	19	19	18	18	18	19	19	28	24	20	19	18	18	16	13	17	21	21	24	21	139	98	26	29	29
			08-7364	####	####	####	####	####	####	####	####	####	23	22	22	22	0	0	0	19	19	18	18	18	19	19	28	24	20	19	18	18	16	13	17	21	21	24	21	139	98	26	29	29
			08-6569	####	####	####	####	####	####	####	####	####	22	21	21	21	19	19	19	0	2	1	1	1	2	4	27	23	7	4	3	5	5	12	16	20	20	23	20	138	97	25	28	28
			08-6997	####	####	####	####	####	####	####	####	####	22	21	21	21	19	19	19	2	0	1	1	1	2	4	27	23	7	4	3	5	5	12	16	20	20	23	20	138	97	25	28	28
	2008 deli meat	1	08-5578	####	####	####	####	####	####	####	####	####	21	20	20	20	18	18	18	1	1	0	0	0	1	3	26	22	6	3	2	4	4	11	15	19	19	22	19	137	96	24	27	27
			08-6056	####	####	####	####	####	####	####	####	####	21	20	20	20	18	18	18	1	1	0	0	0	1	3	26	22	6	3	2	4	4	11	15	19	19	22	19	137	96	24	27	27
			10-0815	####	####	####	####	####	####	####	####	####	21	20	20	20	18	18	18	1	1	0	0	0	1	3	26	22	6	3	2	4	4	11	15	19	19	22	19	137	96	24	27	27
		2	10-0814	####	####	####	####	####	####	####	####	####	22	21	21	21	19	19	19	2	2	1	1	1	0	4	27	23	7	4	3	5	5	12	16	20	20	23	20	138	97	25	28	28
		3	08-7374	####	####	####	####	####	####	####	####	####	22	21	21	21	19	19	19	4	4	3	3	3	4	0	27	23	7	4	3	5	5	12	16	20	20	23	20	138	97	25	28	28
		5	08-7669	####	####	####	####	####	####	####	####	####	31	30	30	30	28	28	28	27	27	26	26	26	27	27	0	32	28	27	26	26	24	21	25	29	29	32	29	147	106	34	37	37
8		6	08-5923	####	####	####	####	####	####	####	####	####	7	6	6	6	24	24	24	23	23	22	22	22	23	23	32	0	24	23	22	22	20	17	19	23	23	26	25	139	98	30	29	29
			98-0291	####	####	####	####	####	####	####	####	####	23	22	22	22	20	20	20	7	7	6	6	6	7	7	28	24	0	7	6	6	6	13	17	21	21	24	21	139	98	26	29	29
			01-1280	####	####	####	####	####	####	####	####	####	22	21	21	21	19	19	19	4	4	3	3	3	4	4	27	23	7	0	3	5	5	12	16	20	20	23	20	138	97	25	28	28
			04-5457	####	####	####	####	####	####	####	####	####	21	20	20	20	18	18	18	3	3	2	2	2	3	3	26	22	6	3	0	4	4	11	15	19	19	22	19	137	96	24	27	27
			99-6370	####	####	####	####	####	####	####	####	####	21	20	20	20	18	18	18	5	5	4	4	4	5	5	26	22	6	5	4	0	4	11	15	19	19	22	19	137	96	24	27	27
			98-2035	####	####	####	####	####	####	####	####	####	19	18	18	18	16	16	16	5	5	4	4	4	5	5	24	20	6	5	4	4	0	9	13	17	17	20	17	135	94	22	25	25
			95-0093	####	####	####	####	####	####	####	####	####	16	15	15	15	13	13	13	12	12	11	11	11	12	12	21	17	13	12	11	11	9	0	10	14	14	17	10	132	91	15	22	22
			88-0478	####	####	####	####	####	####	####	####	####	18	17	17	17	17	17	17	16	16	15	15	15	16	16	25	19	17	16	15	15	13	10	0	16	16	19	18	134	93	23	24	24
	Sporadic		01-5252	####	####	####	####	####	####	####	####	####	22	21	21	21	21	21	21	20	20	19	19	19	20	20	29	23	21	20	19	19	17	14	16	0	6	19	22	138	97	27	28	28
			02-5993	####	####	####	####	####	####	####	####	####	22	21	21	21	21	21	21	20	20	19	19	19	20	20	29	23	21	20	19	19	17	14	16	6	0	19	22	138	97	27	28	28
			88-1059	####	####	####	####	####	####	####	####	####	25	24	24	24	24	24	24	23	23	22	22	22	23	23	32	26	24	23	22	22	20	17	19	19	19	0	25	141	100	30	31	31
			03-5473	####	####	####	####	####	####	####	####	####	24	23	23	23	21	21	21	20	20	19	19	19	20	20	29	25	21	20	19	19	17	10	18	22	22	25	0	140	99	17	30	30
			10-0819	####	####	####	####	####	####	####	####	####	138	137	137	137	139	139	139	138	138	137	137	137	138	138	147	139	139	138	137	137	135	132	134	138	138	141	140	0	169	145	144	144
			11-4254	####	####	####	####	####	####	####	####	####	97	96	96	96	98	98	98	97	97	96	96	96	97	97	106	98	98	97	96	96	94	91	93	97	97	100	99	169	0	104	103	103
			01-1468	####	####	####	####	####	####	####	####	####	29	28	28	28	26	26	26	25	25	24	24	24	25	25	34	30	26	25	24	24	22	15	23	27	27	30	17	145	104	0	35	35
			01-6771	####	####	####	####	####	####	####	####	####	28	27	27	27	29	29	29	28	28	27	27	27	28	28	37	29	29	28	27	27	25	22	24	28	28	31	30	144	103	35	0	6
			99-6871	####	####	####	####	####	####	####	####	####	28	27	27	27	29	29	29	28	28	27	27	27	28	28	37	29	29	28	27	27	25	22	24	28	28	31	30	144	103	35	6	0

#Isolate types: C, Clinical; F, Food; FPE, Food processing environment.

*Isolates origincally included in outbreak investigation due to PFGE patterns that were later excluded due to contrary epidemiological evidence.

Note: Outbreak isolates without epidemiological links to outbreak were omitted from pairwise analysis (Figure 2). Epidemiologically-realted pairs (green); pairs with known no epidemiolgical link belonging to the same CC (blue); pairs with known no epidemiolgical link belonging to a different CC (red);

Table S6. CC8 core hqSNV distance matrix

Outbreak Investigation	Strain (2008 deli meat investigation)	Isolate	10-1321	10-1046	10-1047	10-5024	08-6569	08-6997	08-5578	08-6056	10-0815	10-0814	08-7374	08-7362	08-7363	08-7364	08-7669	08-5923	98-0291	01-1280	04-5457	99-6370	98-2035	95-0093	88-0478	01-1468	01-6771	99-6871	03-5473	88-1059	01-5252	02-5993	11-4254	10-0819
		#Isolate Type	C	C	C	F	FPE	C	C	C	C	C	C	FPE	FPE	FPE	C	C	C	C	C	C	C	C	C	C	C	C	C	C	C	C	C	C
		10-1321	0	1	1	5	24	24	23	23	23	24	24	27	27	27	34	7	27	24	23	23	21	19	18	32	29	29	27	25	23	23	104	147
2010 Prosciutto		10-1046	1	0	0	4	23	23	22	22	22	23	23	26	26	26	33	6	26	23	22	22	20	18	17	31	28	28	26	24	22	22	103	146
		10-1047	1	0	0	4	23	23	22	22	22	23	23	26	26	26	33	6	26	23	22	22	20	18	17	31	28	28	26	24	22	22	103	146
		10-5024	5	4	4	0	23	23	22	22	22	23	23	26	26	26	33	6	26	23	22	22	20	18	17	31	28	28	26	24	22	22	103	146
	1	08-6569	24	23	23	23	0	2	1	1	1	2	4	21	21	21	28	25	9	4	3	5	5	13	18	26	31	31	21	25	23	23	106	149
	1	08-6997	24	23	23	23	2	0	1	1	1	2	4	21	21	21	28	25	9	4	3	5	5	13	18	26	31	31	21	25	23	23	106	149
	1	08-5578	23	22	22	22	1	1	0	0	0	1	3	20	20	20	27	24	8	3	2	4	4	12	17	25	30	30	20	24	22	22	105	148
	1	08-6056	23	22	22	22	1	1	0	0	0	1	3	20	20	20	27	24	8	3	2	4	4	12	17	25	30	30	20	24	22	22	105	148
	1	10-0815	23	22	22	22	1	1	0	0	0	1	3	20	20	20	27	24	8	3	2	4	4	12	17	25	30	30	20	24	22	22	105	148
2008 deli meat	2	10-0814	24	23	23	23	2	2	1	1	1	0	4	21	21	21	28	25	9	4	3	5	5	13	18	26	31	31	21	25	23	23	106	149
	3	08-7374	24	23	23	23	4	4	3	3	3	4	0	21	21	21	28	25	9	4	3	5	5	13	18	26	31	31	21	25	23	23	106	149
		08-7362	27	26	26	26	21	21	20	20	20	21	21	0	0	0	31	28	24	21	20	20	18	16	21	29	34	34	24	28	26	26	109	152
	4	08-7363	27	26	26	26	21	21	20	20	20	21	21	0	0	0	31	28	24	21	20	20	18	16	21	29	34	34	24	28	26	26	109	152
		08-7364	27	26	26	26	21	21	20	20	20	21	21	0	0	0	31	28	24	21	20	20	18	16	21	29	34	34	24	28	26	26	109	152
	5	08-7669	34	33	33	33	28	28	27	27	27	28	28	31	31	31	0	35	31	28	27	27	25	23	28	36	41	41	31	35	33	33	116	159
	6	08-5923	7	6	6	6	25	25	24	24	24	25	25	28	28	28	35	0	28	25	24	24	22	20	19	33	30	30	28	26	24	24	105	148
		98-0291	27	26	26	26	9	9	8	8	8	9	9	24	24	24	31	28	0	9	8	8	8	16	21	29	34	34	24	28	26	26	109	152
		01-1280	24	23	23	23	4	4	3	3	3	4	4	21	21	21	28	25	9	0	3	5	5	13	18	26	31	31	21	25	23	23	106	149
		04-5457	23	22	22	22	3	3	2	2	2	3	3	20	20	20	27	24	8	3	0	4	4	12	17	25	30	30	20	24	22	22	105	148
		99-6370	23	22	22	22	5	5	4	4	4	5	5	20	20	20	27	24	8	5	4	0	4	12	17	25	30	30	20	24	22	22	105	148
		98-2035	21	20	20	20	5	5	4	4	4	5	5	18	18	18	25	22	8	5	4	4	0	10	15	23	28	28	18	22	20	20	103	146
		95-0093	19	18	18	18	13	13	12	12	12	13	13	16	16	16	23	20	16	13	12	12	10	0	13	17	26	26	12	20	18	18	101	144
		88-0478	18	17	17	17	18	18	17	17	17	18	18	21	21	21	28	19	21	18	17	17	15	13	0	26	25	25	21	19	17	17	100	143
Sporadic		01-1468	32	31	31	31	26	26	25	25	25	26	26	29	29	29	36	33	29	26	25	25	23	17	26	0	39	39	17	33	31	31	114	157
		01-6771	29	28	28	28	31	31	30	30	30	31	31	34	34	34	41	30	34	31	30	30	28	26	25	39	0	8	34	32	30	30	111	154
		99-6871	29	28	28	28	31	31	30	30	30	31	31	34	34	34	41	30	34	31	30	30	28	26	25	39	8	0	34	32	30	30	111	154
		03-5473	27	26	26	26	21	21	20	20	20	21	21	24	24	24	31	28	24	21	20	20	18	12	21	17	34	34	0	28	26	26	109	152
		88-1059	25	24	24	24	25	25	24	24	24	25	25	28	28	28	35	26	28	25	24	24	22	20	19	33	32	32	28	0	20	20	107	150
		01-5252	23	22	22	22	23	23	22	22	22	23	23	26	26	26	33	24	26	23	22	22	20	18	17	31	30	30	26	20	0	6	105	148
		02-5993	23	22	22	22	23	23	22	22	22	23	23	26	26	26	33	24	26	23	22	22	20	18	17	31	30	30	26	20	6	0	105	148
		11-4254	104	103	103	103	106	106	105	105	105	106	106	109	109	109	116	105	109	106	105	105	103	101	100	114	111	111	109	107	105	105	0	183
		10-0819	147	146	146	146	149	149	148	148	148	149	149	152	152	152	159	148	152	149	148	148	146	144	143	157	154	154	152	150	148	148	183	0

#Isolate types: C, Clinical; F, Food; FPE, Food processing environment.

*Isolates origincally included in outbreak investigation due to PFGE patterns that were later excluded due to contrary epidemiological evidence.

Note: Outbreak isolates without epidemiological links to outbreak were omitted from pairwise analysis (Figure 2). Epidemiologically-realted pairs (green); pairs with known no epidemiolgical link belonging to the same CC (blue); pairs with known no epidemiolgical link belonging to a different CC (red);

Table S7. Publically *Listeria monocytogenes* genomes

Strain	Year Isolated	Strain Background	Location	Specimen type	Source	Serotype	MLST	Clonal Comple	Epidemiology	Accession	Reference
F2365	1985	Mexican style soft cheese outbreak, 1985	California, USA	Food	Cheese	4b	1	1	142 cases including 48 deaths (20 in utero, 10 neonates, 18 adults)	AE017262	[4,5]
FSL N1-017				Food	Trout in brine	4b	3	3	Not associated with human disease	AARP00000000	[4,6]
Clip80459	1999	Jellied pork tongue outbreak, 1999	France	Human		4b	4	4	32 cases: CNS listeriosis, bacteremia, 6 deaths (5 adult, 1 in utero), 7 premature births	FM242711.1	[4,7]
HPB2262 (Aureli1997)	1997	Corn salad outbreak, 1997	Northern Italy	Human	Corn salad (from 2 School cafeterias)	4b	2	2	1700 cases including 292 hospitalized (febrile gastrointeritis, mostly children)	AATL00000000	[4,8]
ScottA	1983	Pasteurized milk outbreak, 1983	Massachusetts, USA	Human		4b	2	2	49 cases: 7 fetus/infant, 42 adult, 14 deaths	CM001159	[4,9,10]
FSL R2-503 (G6054)	1994	Sporadic febrile gastroenteritis clinical case associated with chocolate milk, 1994	Illinois, USA	Human	Stool	1/2b	3	3	45 cases including 4 hospitalizations, 0 deaths	AARR00000000	[4,11,12]
FSL J1-194		Sporadic clinical case	USA	Human	Unknown	1/2b	88		Sporadic	AARJ00000000	[4,11]
FSL J1-175				Environment	Water	1/2b	87		Environmental isolate, not associated with disease	AARK00000000	[4,11]
FSL J2-064			USA	Human		1/2b	5	5		CP006592	[4,6]
10403S	1987	Lab derived strain	USA	Mouse		1/2a	85	7	Common lab strain, streptomycin resistant derivative of strain 10403 isolated from human skin lesion in 1968 at Montana State University	CP002002	[4,13]
EGD	1924		UK	Lab strain		1/2a	12	7	Derived from original strain isolated from rabbits by E.G.D. Murray	HG421741	[4,14]
EGD-e	1960		France	Lab strain	Unknown	1/2a	35	9	EGD obtained from Trudeau institute	AL591824	[4,5]
J2818	2000	RTE turkey deli meat outbreak, 2000	USA	Food	RTE turkey	1/2a	86		Processed turkey outbreak in 11 states sourced to single processing plant	AARX00000000	[4,15]
J0161 (FSL R2-499)	2000			Human		1/2a	11			CP002001	
F6900	1989	Sporadic clinical case, 1989	USA	Human		1/2a	86		Linked to processed meat from same facility as J0161 & J2818 isolates 11 yrs later	AARU00000000	[11]
FSL N3-165			USA	Environment	Soil	1/2a	90	90	Not associated with disease	AARQ00000000	[4,16]
Finland 1998	1999	Dairy butter outbreak, 1999	Finland			3a			25 cases including 20 sepsis, 4 meningitis, 1 abcess; 6 deaths	CP002004	[11,17]
FSL R2-561						1/2c				CP002003	[11]

LO28	<1985	<1985		Human	Stool	1/2c	210	9	Feces of healthy, pregnant clinical case	AARY00000000	[4,11,18]
FSL J1-208	1998	Farm animal outbreak, 1998	Georgia, USA	Animal	Caprine brain	4a				CM001469/CM001470	[11,19]
HCC23	2000	2000		Fish	Catfish brain	4a			Naturally avirulent	CP001175	[20]
L99	1950s	1950's	Netherlands	Food		4a			Weakly pathogenic to humans	FM211688	[21,22]
FSL J2-071		Clinical animal case		Animal		4c	131	71		AARN00000000	[11]

Table S8. Sequences of substituted primers used for MLST

Primer	Sequence
*NMLcat-R**	CAGGAAACAGCTATGACCGCTTGCTCCGTCGAAAGGTTC
*NMLdapE-R**	CAGGAAACAGCTATGACCATCGAACTATGGGSATTTTTACC
*NMLdat-R**	CAGGAAACAGCTATGACCTGCGTCCATAATACACCRTCTTT
*NMLldh-F**	GTAAAACGACGGCCAGTGGATTTRAGCCATGCCGTTCC
*NMLldh-R**	CAGGAAACAGCTATGACCACRCCGTAGAATGTAGCGCC
*NMLlhkA-F**	GTAAAACGACGGCCAGTCAAAGCCAAGTAGATGACTTAAC
lhk-R2#	CAGGAAACAGCTATGACCTACGCATTTCATGAGAAACATCAG

* Primers for MLST were modified from Ragon et al. [1], based on an alignment of the following *L. monocycotgenes* genomes (*n*=16): (08-5578, 08-5923, 08-6997, 08-7669, 10-0933, 10-1046, 10-4754, 10-4758, 10-5026, 81-0861, 98-2035, 99-6370, Clip81459, EGD-e, F2365, HCC23) at the seven MLST loci. Refer to **Tables S1** and **S2** for accession numbers. Specifically, the *cat* reverse primer, *ldh* forward and reverse primers, and the *lhkA* forward primer were redesigned, the *dapE* reverse and *dat* reverse primers had degenerate base substitutions.

lhkA-R2 (www.pasteur.fr/mlst) was used to replace the *lhkA* reverse primer

Supplemental References

[1] **Ragon M, Wirth T, Hollandt F, Lavenir R, Lecuit M, Le Monnier A, et al.** A new perspective on Listeria monocytogenes evolution. *PLoS Pathog* 2008 Sep 5;4(9):e1000146.

[2] **Larkin MA, Blackshields G, Brown NP, Chenna R, McGettigan PA, McWilliam H, et al.** Clustal W and Clustal X version 2.0. *Bioinformatics* 2007 Nov 1;23(21):2947-2948.

[3] **Darling AE, Mau B, Perna NT.** progressiveMauve: multiple genome alignment with gene gain, loss and rearrangement. *PLoS One* 2010 Jun 25;5(6):e11147.

[4] **Cantinelli T, Chenal-Francisque V, Diancourt L, Frezal L, Leclercq A, Wirth T, et al.** "Epidemic clones" of Listeria monocytogenes are widespread and ancient clonal groups. *J Clin Microbiol* 2013 Nov;51(11):3770-3779.

[5] **Glaser P, Frangeul L, Buchrieser C, Rusniok C, Amend A, Baquero F, et al.** Comparative genomics of Listeria species. *Science* 2001 Oct 26;294(5543):849-852.

[6] **Gray MJ, Zadoks RN, Fortes ED, Dogan B, Cai S, Chen Y, et al.** Listeria monocytogenes isolates from foods and humans form distinct but overlapping populations. *Appl Environ Microbiol* 2004 Oct;70(10):5833-5841.

[7] **de Valk H, Vaillant V, Jacquet C, Rocourt J, Le Querrec F, Stainer F, et al.** Two consecutive nationwide outbreaks of Listeriosis in France, October 1999-February 2000. *Am J Epidemiol* 2001 Nov 15;154(10):944-950.

[8] **Aureli P, Fiorucci GC, Caroli D, Marchiaro G, Novara O, Leone L, et al.** An outbreak of febrile gastroenteritis associated with corn contaminated by Listeria monocytogenes. *N Engl J Med* 2000 Apr 27;342(17):1236-1241.

[9] **Briers Y, Klumpp J, Schuppler M, Loessner MJ.** Genome sequence of Listeria monocytogenes Scott A, a clinical isolate from a food-borne listeriosis outbreak. *J Bacteriol* 2011 Aug;193(16):4284-4285.

[10] **Fleming DW, Cochi SL, MacDonald KL, Brondum J, Hayes PS, Plikaytis BD, et al.** Pasteurized milk as a vehicle of infection in an outbreak of listeriosis. *N Engl J Med* 1985 Feb 14;312(7):404-407.

[11] **Wiedmann M, Swaminathan B, Lauer P, Portnoy D, Cossart P, Buchrieser C, et al.** Listeria monocytogenes Database. Available at: http://www.broadinstitute.org/annotation/genome/listeria_group/GenomeDescriptions.html. Accessed 09/25, 2015.

[12] **Dalton CB, Austin CC, Sobel J, Hayes PS, Bibb WF, Graves LM, et al.** An outbreak of gastroenteritis and fever due to Listeria monocytogenes in milk. *N Engl J Med* 1997 Jan 9;336(2):100-105.

[13] **Bishop DK, Hinrichs DJ.** Adoptive transfer of immunity to Listeria monocytogenes. The influence of in vitro stimulation on lymphocyte subset requirements. *J Immunol* 1987 Sep 15;139(6):2005-2009.

[14] **Becavin C, Bouchier C, Lechat P, Archambaud C, Creno S, Gouin E, et al.** Comparison of widely used Listeria monocytogenes strains EGD, 10403S, and EGD-e highlights genomic variations underlying differences in pathogenicity. *MBio* 2014 Mar 25;5(2):e00969-14.

[15] **Olsen SJ, Patrick M, Hunter SB, Reddy V, Kornstein L, MacKenzie WR, et al.** Multistate outbreak of Listeria monocytogenes infection linked to delicatessen turkey meat. *Clin Infect Dis* 2005 Apr 1;40(7):962-967.

[16] **Nightingale KK, Schukken YH, Nightingale CR, Fortes ED, Ho AJ, Her Z, et al.** Ecology and transmission of Listeria monocytogenes infecting ruminants and in the farm environment. *Appl Environ Microbiol* 2004 Aug;70(8):4458-4467.

[17] **Lyytikainen O, Autio T, Maijala R, Ruutu P, Honkanen-Buzalski T, Miettinen M, et al.** An outbreak of Listeria monocytogenes serotype 3a infections from butter in Finland. *J Infect Dis* 2000 May;181(5):1838-1841.

[18] **Vicente MF, Baquero F, Pérez-Diaz JC.** Cloning and expression of the *Listeria monocytogenes* haemolysin in *Escherichia coli*. *FEMS Microbiology Letters* 1985;30(1-2):77–79.

[19] **den Bakker HC, Bowen BM, Rodriguez-Rivera LD, Wiedmann M.** FSL J1-208, a virulent uncommon phylogenetic lineage IV Listeria monocytogenes strain with a small chromosome size and a putative virulence plasmid carrying internalin-like genes. *Appl Environ Microbiol* 2012 Mar;78(6):1876-1889.

[20] **Steele CL, Donaldson JR, Paul D, Banes MM, Arick T, Bridges SM, et al.** Genome sequence of lineage III Listeria monocytogenes strain HCC23. *J Bacteriol* 2011 Jul;193(14):3679-3680.

[21] **Hain T, Ghai R, Billion A, Kuenne CT, Steinweg C, Izar B, et al.** Comparative genomics and transcriptomics of lineages I, II, and III strains of Listeria monocytogenes. *BMC Genomics* 2012 Apr 24;13:144-2164-13-144.

[22] **Kuenne C, Billion A, Mraheil MA, Strittmatter A, Daniel R, Goesmann A, et al.** Reassessment of the Listeria monocytogenes pan-genome reveals dynamic integration hotspots and mobile genetic elements as major components of the accessory genome. *BMC Genomics* 2013 Jan 22;14:47-2164-14-47.
